# A Miniaturized Electrochemical SERS Chip with 3D Nanoporous Gold Interface for Qualitative and Quantitative Analysis of Organic Pollutants and their Oxidation Intermediates

**DOI:** 10.1002/smll.202503894

**Published:** 2025-07-23

**Authors:** Xu Wang, Yong Yan, Huan Zhou, Xinyu Liu, Riyi Zhang, Dong Wang, Peter Schaaf, Guangsheng Guo, Xiayan Wang

**Affiliations:** ^1^ State Key Laboratory of Materials Low‐Carbon Recycling Center of Excellence for Environmental Safety and Biological Effects Department of Chemistry College of Chemistry and Life Science Beijing University of Technology Beijing 100124 P. R. China; ^2^ Center for Nanochemistry Beijing Science and Engineering Center for Nanocarbons Beijing National Laboratory for Molecular Sciences College of Chemistry and Molecular Engineering Peking University Beijing 100871 P. R. China; ^3^ Chair Materials for Electrical Engineering and Electronics Institute of Materials Science and Engineering and Institute of Micro‐ and Nanotechnologies MarcoNano TU Ilmenau Gustav‐Kirchhoff‐Str. 5 98693 Ilmenau Germany

**Keywords:** 3D nanoporous Au‐Ag (3D Au‐Ag NPs), electrochemical surface‐enhanced Raman scattering (EC‐SERS), microfluidic chip

## Abstract

Polycyclic aromatic hydrocarbons (PAHs) and organophosphorus pesticides (OPPs) are typical pollutants characterized by a wide variety of types, difficulty in degradation, and significant differences in mutagenic and carcinogenic toxicity. However, due to their distinct physicochemical properties, it remains challenging to simultaneously analyze PAHs and OPPs under real environmental conditions within the same simplified detection systems and accurately assess their oxidation‐reduction processes. In this study, electrochemical surface‐enhanced Raman scattering (EC‐SERS), utilizing a microfluidic chip of self‐made 3D nanoporous Au loaded with Ag nanoparticles, is employed to analyze PAHs and OPPs in the environment and investigate their oxidation‐reduction processes, which displayed excellent sensitivity and selectivity. The chip successfully analyzed three types of PAHs and four types of OPPs, achieving the lowest detection limit of 0.01 ppb, and strong linearity within a certain range. The recovery in real water samples reached 102.89% with a relative standard deviation (RSD) of 13.36%. Furthermore, this system successfully identified different types of pollutants and their concentrations in mixed samples with high accuracy. Meanwhile, preliminary insights into the electrochemical oxidation and degradation processes of these pollutants are provided, offering a first understanding of their transformation mechanisms.

## Graphical abstract

A 3D continuous Au/Ag bimetallic synergistic interface is constructed on a microfluidic chip, and high‐sensitivity detection and oxidative pathway elucidation of polycyclic aromatic hydrocarbons (PAHs) and organophosphorus pesticides (OPPs) in environmental samples are achieved by coupling with electrochemical surface‐enhanced Raman scattering (EC‐SERS) technology. Based on the EC‐SERS microfluidic platform with an Au/Ag bimetallic synergistic interface, the simultaneous detection of chemically distinct pollutants (PAHs and OPPs) is enabled for the first time at trace levels (as low as 0.01 ppb) by integrating a hydrophobic‐hydrophilic cooperative enrichment mechanism with dynamic electrochemical potential modulation. This strategy overcame the limitations of conventional methods and allowed real‐time in situ tracking of electrochemical oxidation pathways, providing a highly integrated platform for precise monitoring and mechanistic studies of complex environmental contaminants.

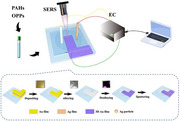

## 1 Introduction

Priority pollutants (PPs) are toxic contaminants that threaten human health and ecosystems, necessitating urgent detection and control.^[^ ^1^ ^]^ Among these, persistent pollutants, most notably polycyclic aromatic hydrocarbons (PAHs) and organophosphorus pesticides (OPPs), have garnered significant attention.^[^ ^2^ ^]^ PAHs are hydrophobic compounds with multiple fused aromatic rings, originating from natural sources (e.g., volcanic eruptions, forest fires) and human activities (e.g., incomplete combustion of fossil fuels), and exhibit potential carcinogenic risks due to their strong bioaccumulation.^[^ ^3^ ^]^ In contrast, OPPs are widely used hydrophilic pesticides that irreversibly phosphorylate acetylcholinesterase, leading to neurotoxicity, respiratory damage, or even death.^[^ ^4^ ^]^ It should be noted that, even within the similar type of PAHs or OPPs, variations in molecular structure and concentration can yield vastly different toxicity profiles, underscoring the need for accurate qualitative and quantitative detection and making rapid, reliable on‐site detection of these pollutants crucial for safeguarding food, water, and environmental safety.^[^ ^5^, ^6^ ^]^

Currently, the most common techniques for detecting PAHs and OPPs, such as infrared spectroscopy, liquid chromatography, gas chromatography, and liquid chromatography–mass spectrometry, each have distinct advantages but are typically hindered by complex, time‐consuming, and labor‐intensive protocols, along with high costs and difficulties in miniaturization. Moreover, the need to simultaneously detect these two pollutants, which exhibit markedly different physicochemical properties, further complicates the detection process. ^[^ ^7^, ^8^, ^9^ ^]^

Surface‐enhanced Raman spectroscopy (SERS) has emerged as a powerful ultra‐sensitive technique for analyzing environmental pollutants due to its rich molecular fingerprint information, simplicity, and high detection efficiency.^[^ ^10^ ^]^ By confining target molecules within plasmonic “hotspots”, SERS can achieve single‐molecule sensitivity.^[^ ^11^, ^12^ ^]^ Recently, electrochemical surface‐enhanced Raman scattering (EC‐SERS) has further enhanced this approach, allowing researchers to modulate and distinguish the redox states of analytes, thereby enabling both qualitative and quantitative monitoring of structurally similar molecules.^[^ ^13^ ^]^ PAHs and OPPs have drawn growing interest in SERS‐based detection, given their distinct molecular vibrations and toxicological relevance.^[^ ^14^ ^]^ The key to SERS lies in localized surface plasmon resonance (LSPR) generated by noble metal materials (e.g., Au and Ag), which significantly amplifies electromagnetic fields near their surfaces.^[^ ^15^, ^16^ ^]^ Although various Au/Ag nanostructures have been employed for detecting environmental pollutants, issues such as nanoparticle aggregation and uneven hotspot distribution often restrict their reproducibility and sensitivity.^[^ ^17^ ^]^ Moreover, establishing an integrated and robust EC‐SERS substrate/electrode remains challenging, especially for simultaneously detecting pollutants with widely differing properties (like PAHs and OPPs). Thus, developing a stable, uniform, and highly efficient EC‐SERS platform is essential for accurate on‐site analysis and real‐time monitoring of these priority pollutants.

In this study, a miniaturized EC‐SERS chip featuring a 3D continuous nanoporous Au microfluidic interface was prepared through a combination process of magnetron sputtering and dealloying. This chip demonstrated an excellent performance in simultaneously detecting polycyclic aromatic hydrocarbons (PAHs) and organophosphorus pesticides (OPPs) under real environmental conditions and accurately examining their redox processes. With further modification of Ag components, the 3D sponge‐like Au substrate not only provides uniform, high‐density “hotspots” for SERS enhancement but also serves as an integrated working electrode, which showed the relative lowest detection limits of 0.01 ppb and 0.01 ppb for PAHs and OPPs, respectively, and demonstrating linearity over a certain concentration range (0.03‐124 ppb) and a recovery of up to 102.89% in actual water samples with a relative standard deviation (RSD) of 13.36%. This EC‐SERS chip analysis system demonstrated excellent uniformity, versatility, and practicality, with the chip showing strong potential for applications in biosensing, environmental monitoring, and pollutant degradation.

## 2 Results and Discussion

### 2.1 Morphologies and Performance of the 3D SERS Substrate

**Figure 1:**
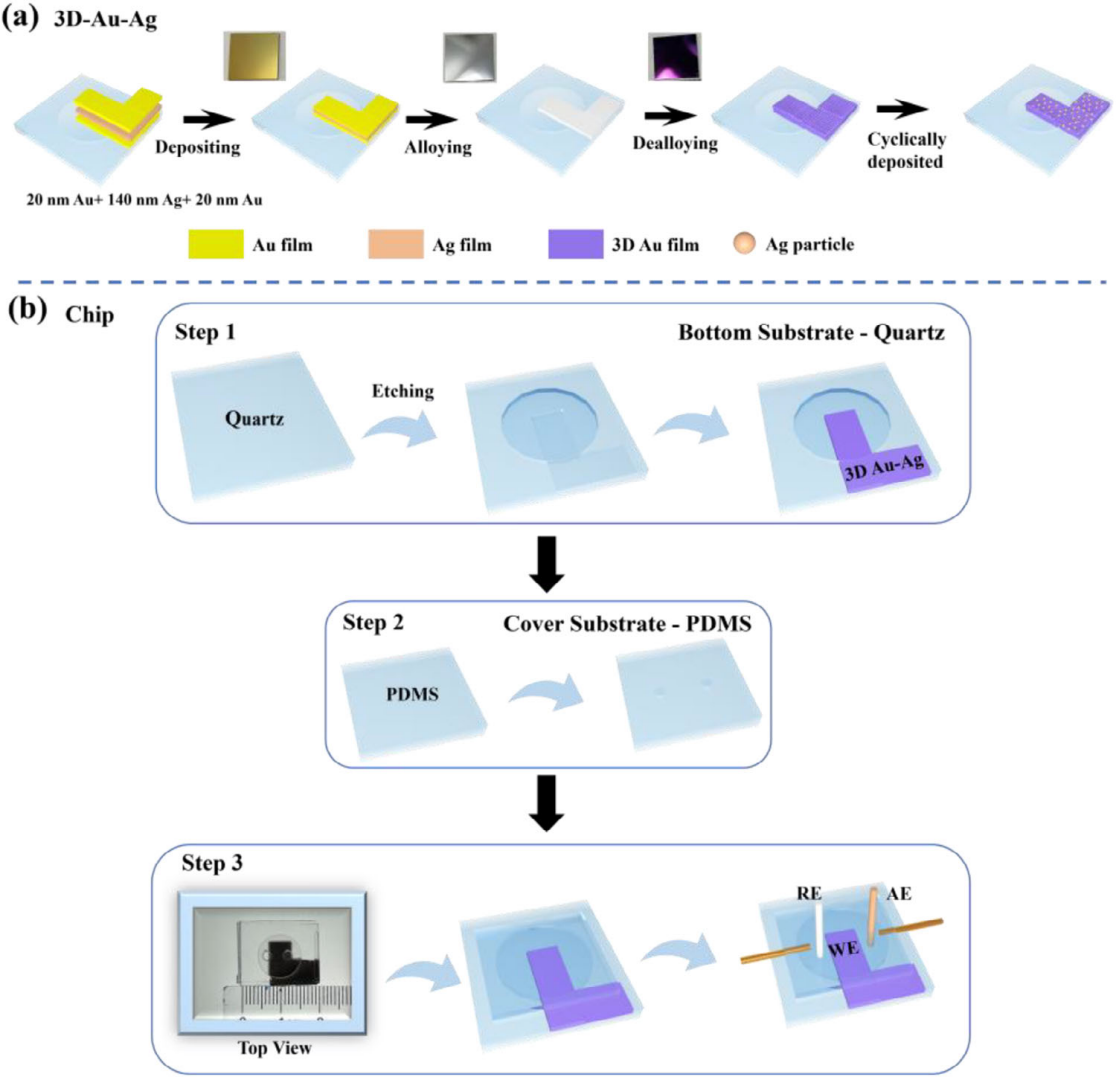
Process flow diagram for preparing 3D‐Au‐Ag a); Process flow diagram and physical image of the assembled chip b).

**Figure 2:**
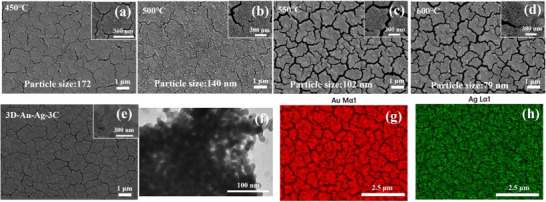
The SEM images of 3D‐Au substrate obtained by alloying annealing at a) 450 °C, b) 500 °C, c) 550 °C, and d) 600 °C; e) the SEM image of 3D‐Au‐Ag‐3C substrate prepared with 3D‐Au (alloying annealing at 500 °C) as precursor; f) TEM image and g, h) EDS results of 3D‐Au‐Ag substrate.

A 3D porous Au framework (3D‐Au) was fabricated via a alloying‐dealloying processes (details were provided in the experimental section, **Figure** **1**a). The fabricated 3D‐Au film was composed of interconnected Au nanoribbons, forming a 3D continuous nanoporous structure. When the alloy annealing temperature exceeded 600 °C or fell below 450 °C, a significant number of fractures appeared within the 3D‐Au film, severely affecting the uniform diffusion at the SPR interface. This could potentially lead to increased random errors in SERS detection and limited reproducibility, as illustrated in Figure S2a,b (Supporting Information). The alloying annealing temperature also significantly affected the Au nanoribbons size. Based on the grayscale gradient method, the endpoints of the 3D Au nanoribbons were manually identified from the SEM images (**Figure** **2**a–d) for statistical analysis. The endpoints were defined as positions exhibiting abrupt variations in brightness or contrast. Nanoribbon fragments with significantly abnormal widths were excluded. For each SEM image, five randomly selected regions were analyzed, and a total of 100 nanoribbons were measured to ensure the statistical randomness of the dataset. The measured lengths were compiled and presented as histograms illustrating the nanoribbon length distribution (Figure S2c–f, Supporting Information). After statistics, their average width reduced from 172 to 79 nm with the annealing temperature increased from 450 to 600 °C (Figure 2a–d; Figure S2c–f, Supporting Information).

Additionally, Ag nanoparticles were controllably deposited on the surface of the prepared 3D‐Au film through a wet chemical cyclic Ag plating method. The characterization results of the prepared 3D Au‐Ag NPs are shown in Figure 2 and Figure S14 (Supporting Information). As reported in another study, the Ag‐Au bimetallic interface exhibited higher sensitivity in SERS detection compared to the pure Au interface, particularly for hydrophobic analytes.^[^ ^18^, ^19^ ^]^ To further elucidate the field enhancement mechanism of the 3D‐Au, a finite‐difference time‐domain (FDTD) simulation was conducted using a microstructural model with dimensions of 300 × 300 nm. As shown in Figure S15 (Supporting Information), electromagnetic field distributions were simulated at four characteristic excitation wavelengths derived from the UV–Vis–NIR absorption spectrum. The results revealed a pronounced increase in the local electric field enhancement factor as the incident light wavelength shifted from the ultraviolet to the near‐infrared region. Notably, the high‐intensity plasmonic “hotspots” observed in the electromagnetic field distribution maps (indicated by the bright regions) exhibited strong spatial correlation with the surface morphology of the 3D mesoporous framework. This finding indicated that the 3D topological architecture of mesoporous gold effectively facilitated synergistic surface plasmon resonance enhancement under long‐wavelength excitation.

### 2.2 Fabrication and Performance Evolution of an EC‐SERS Detection System Based on 3D Au‐Ag NPs Chip

The EC‐SERS detection chip consisted of a bottom quartz substrate and a top PDMS substrate. The bottom quartz substrate featured a detection cell with a 0.5 cm radius and a 3D Au‐Ag thin film, while the top PDMS cover had two circular channels with a 0.1 cm radius to facilitate the insertion of a platinum counter electrode and a reference electrode. Finally, the quartz substrate with integrated electrodes was assembled with the PDMS substrate to fabricate the 3D Au‐Ag NPs SERS chip, as shown in the optical image in Figure 1b.

The electrochemical performance of the constructed EC‐SERS chip was subsequently evaluated, with the results presented in Figure S3 (Supporting Information). The electrochemical signal intensity increased with the concentration of K_3_[Fe(CN)_6_]/K_4_[Fe(CN)_6_], exhibiting a linear relationship within the concentration range of 1.25‐80 mmol L^−1^. The corresponding linear regression equation was determined as *y* = 0.09160 *x* + 0.08350 with a coefficient of determination *R^2^* = 0.9939. Additionally, the experimental results demonstrated excellent reproducibility, with a RSD of 7.2%, confirming the robust electrochemical detection performance of the three‐electrode system. Moreover, additional experiments were performed to further assess signal reproducibility. The prepared substrates were used to detect pyrene and fenitrothion (50 ppm), with 100 repeated measurements conducted across different regions of the same substrate as well as among different substrates. As shown in Figure S16 (Supporting Information), the RSDs were determined to be 10.85% and 1.55%, respectively. These results confirmed that, despite localized morphological fluctuations at the nanoscale, both the nanoparticle density and Raman signal reproducibility at the operational scale were sufficient to support reliable quantitative analysis.

### 2.3 Method Verification and Real Sample Analysis

**Figure 3:**
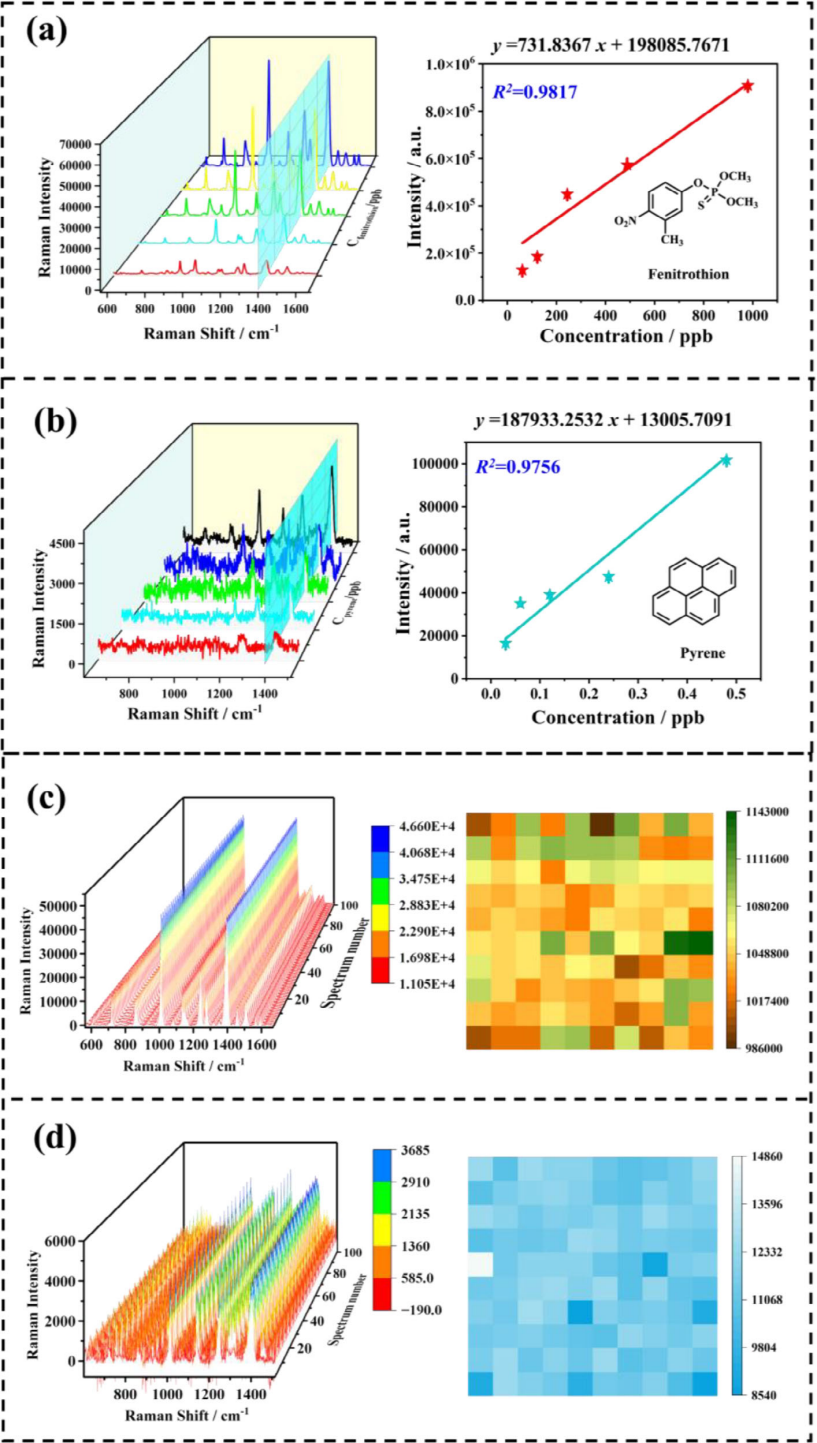
a) SERS spectra of fenitrothion (61‐980 ppb) with the corresponding linear relationship based on the logarithmic intensity at 1015 cm⁻¹; b) SERS spectra of pyrene (0.03‐0.48 ppb) with the corresponding linear relationship based on the logarithmic intensity at 1408 cm⁻¹; c) Stability test results of 100 samples of fenitrothion at a concentration of 490 ppb; d) Stability test results of 100 samples of pyrene at a concentration of 0.06 ppb.

A systematic methodological validation was conducted in this study to comprehensively evaluate the linear range, precision, stability, limit of detection (LOD), and limit of quantification (LOQ) of the proposed detection system. The construction of calibration curves based on concentration versus peak area (mean values from triplicate measurements) effectively minimized systematic experimental errors. Notably, the method exhibited a distinct dual‐linear response characteristic: the direct solution‐dropping approach demonstrated good linearity (*R^2^* > 0.95) within the low concentration range (0.01‐980 ppb), while the solvent evaporation‐induced molecular aggregation strategy extended the detection capability to higher concentrations (3.91‐500 ppm), significantly broadening the dynamic range. Sensitivity assessments revealed that the LOD (S/N = 3) for OPPs ranged from 0.01 to 37.20 ppb, with profenofos achieving an ultra‐sensitive detection level (LOD = 0.01 ppb) and fenitrothion reaching 37.20 ppb in **Figure** **3**a (complete data provided in Figures S4–S6, Supporting Information). For PAHs, pyrene exhibited an LOD of 0.01 ppb (Figure 3b), while high‐molecular‐weight PAHs demonstrated superior detection sensitivity, likely due to their larger Raman scattering cross‐sections enhancing signal intensity (Figures S7–S8, Supporting Information). The LOQs (S/N = 10) met the requirements for trace analysis (Table S1, Supporting Information). Through this methodological validation, the dual‐mode semi‐quantitative detection of PAHs and organophosphate pesticides in environmental contaminants was successfully achieved.

To evaluate the stability of the proposed method, a stability assessment based on 100 consecutive repeated measurements was conducted. The results demonstrated excellent analytical reproducibility across different concentration gradients and pollutant types. As shown in Figure 3c,d, at low concentrations (0.01–1 ppb), the RSD for anthracene (0.24 ppb, RSD = 4.33%, Figure S8, Supporting Information) and pyrene (0.06 ppb, RSD = 7.61%) remained below 8%. At higher concentration levels (100–500 ppb), the RSDs for naphthalene (490 ppb, RSD = 3.81%, Figure S7, Supporting Information), fenitrothion, and isocarbophos (both RSD = 2.73%), as well as omethoate (320 ppb, RSD = 3.43%), were all below 5% (Figures S4–S5, Supporting Information). Meanwhile, in the moderate concentration range, profenofos exhibited an RSD of 5.61% (Figure S6, Supporting Information), which still met the requirements for trace analysis (RSD < 10%). These results systematically validated the reliability of this method for detecting multiple classes of pollutants across a broad concentration range (0.01‐500 ppb), thereby providing a robust analytical foundation for practical sample analysis.

**Figure 4:**
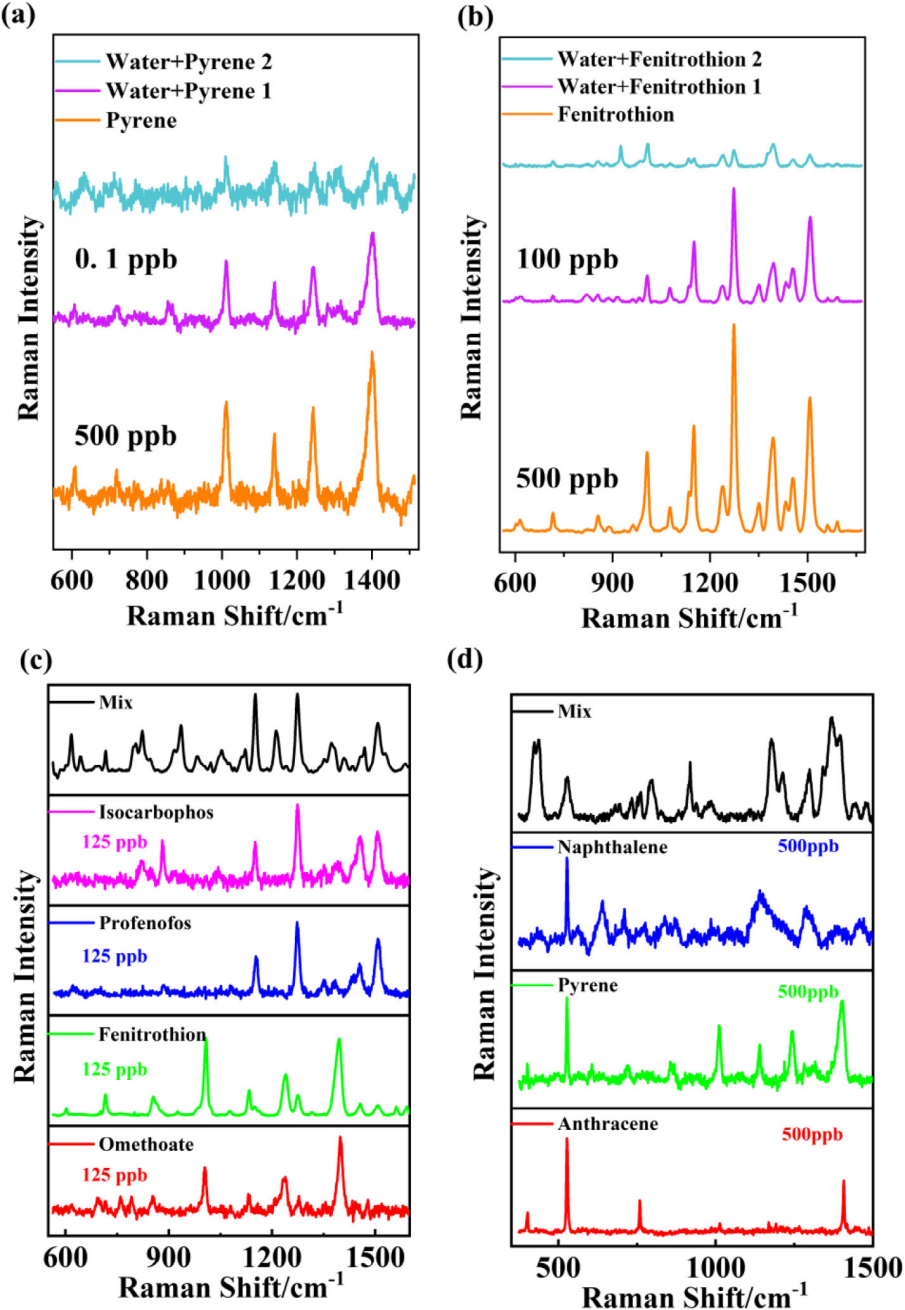
Spiked recovery results of pyrene in real water samples a); Spiked recovery results of fenitrothion in real water samples b); Raman spectra of three PAHs mixture c); Raman spectra of four OPPs mixture d).

To evaluate the effectiveness and applicability of the 3D‐Au‐Ag substrate, this SERS platform was applied to the detection of pyrene and fenitrothion in real water samples. These two pollutants were selected due to their relatively high toxicity and widespread occurrence among PAHs and organophosphorus pesticides. Water samples from Baiyangdian Lake (freshwater lake in Hebei Province, China) were chosen as real samples. Since no target contaminants were detected in the blank samples, spiked concentrations of 500 ppb, 100 ppb, and 0.1 ppb were selected based on the detection levels specified in the safe water standards. The accuracy of this method was validated by determining the spiked recovery and RSD (**Figure** **4**a,b). The results showed that spiked recoveries ranged from 93.68% to 102.89%, with RSD values below 13.36%. Impurities present in the real water samples did not cause noticeable matrix effects, demonstrating the 3D‐Au‐Ag substrate's high effectiveness and applicability for environmental pollutant detection. Moreover, when testing mixed samples of similar pollutants, despite slight intensity changes and peak shifts due to molecular interactions at the SERS interface, the SERS spectra still enabled reliable identification of different pollutant molecules (Figure 4c,d).

### 2.4 In‐Situ EC‐SERS Detection of PAHs and OPPs

Due to the high structural similarity of certain pollutant molecules, such as PAHs, their Raman fingerprint signals often exhibited significant overlap. Additionally, organic extraction solvents and electrolytes suppressed or obscured characteristic peaks, further complicating molecular identification. Moreover, some pollutants potentially generated more toxic intermediates during degradation, making it essential to elucidate their degradation kinetics. The redox signals generated during oxidation effectively distinguished structurally similar molecules. To address this challenge, in situ electrochemical surface‐enhanced Raman spectroscopy (EC‐SERS) was employed in this study. By applying specific reaction potentials, pollutants adsorbed on the SERS substrate were detected, enabling further exploration of secondary molecular fingerprint information associated with oxidation/reduction states. This approach provided a novel method for precise molecular identification and degradation mechanism investigation.

**Figure 5:**
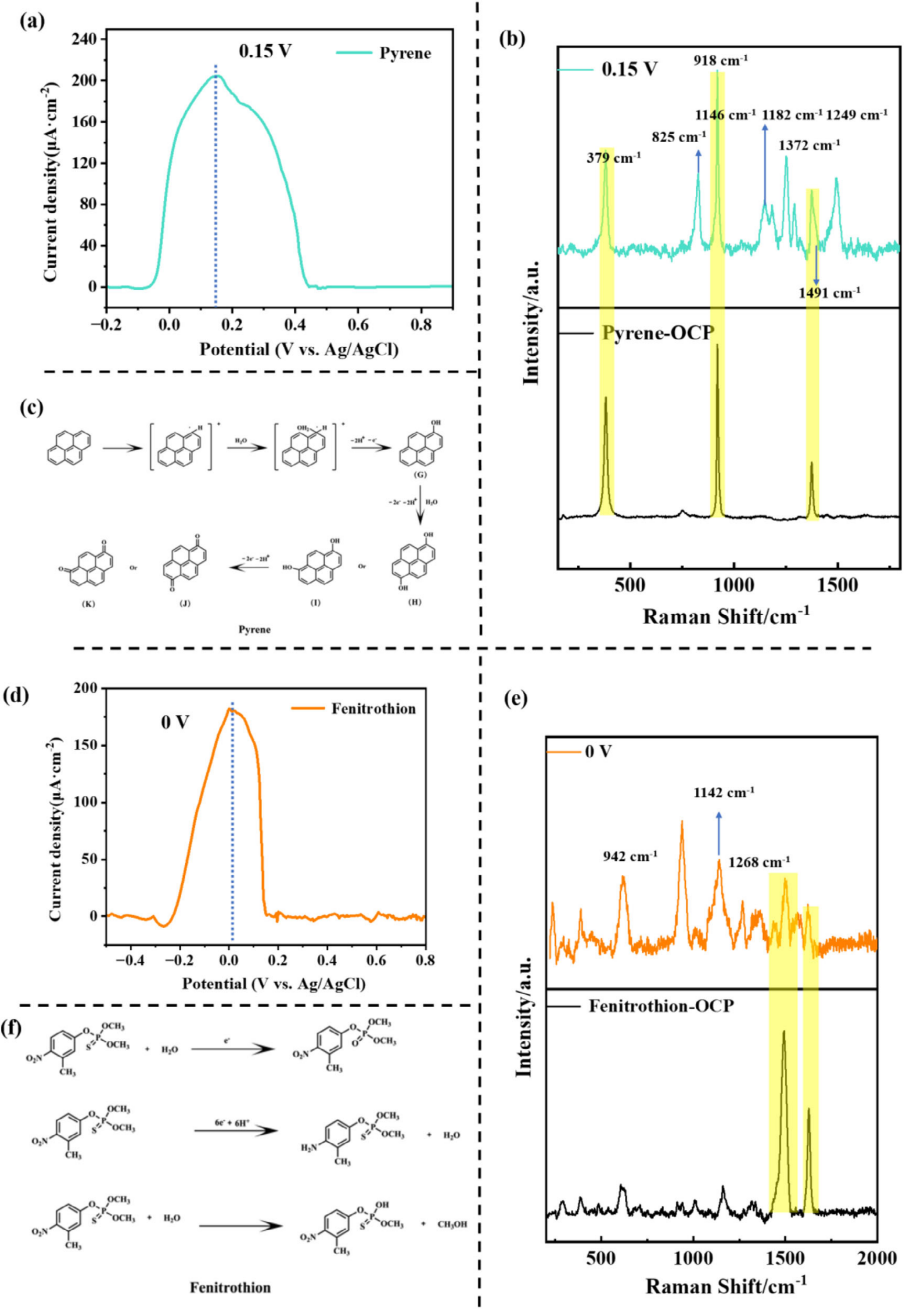
Selective analysis of 50 ppb PAHs using the Microchip EC‐SERS detection method at applied electrochemical potentials: a) DPV curve of pyrene; b) Raman spectrum of pyrene; c) proposed oxidation mechanism of pyrene; Selective analysis of 50 ppb OPPs using the Microchip EC‐SERS detection method at applied electrochemical potentials: d) DPV curve of fenitrothion; e) Raman spectrum of fenitrothion; f) proposed oxidation mechanism of fenitrothion.

Based on the differential pulse voltammetry (DPV) characterization results (**Figure** **5**a), pyrene exhibited a characteristic oxidation peak at +0.15 V (versus Ag/AgCl). Under this optimized potential, in situ electrochemical surface‐enhanced Raman spectroscopy (EC‐SERS) detection was performed, revealing a significant enhancement of characteristic peaks at 825 cm⁻¹ (C‐H out‐of‐plane bending), 1146 cm⁻¹ (C‐C symmetric stretching), 1182 cm⁻¹ (ring‐breathing vibration), 1249 cm⁻¹ (C‐H in‐plane bending), and 1491 cm⁻¹ (C═C skeletal vibration) (Figure 5b).^[^ ^20^, ^21^ ^]^ Prior to each measurement, the working electrode was pre‐scanned over an extended potential window using the electrolyte solution. This procedure effectively removed any residual species, and no interference was observed within the target potential range. Subsequent measurements of other analytes were then carried out without cross‐contamination. Notably, this experimental approach also demonstrated distinct spectral differences for naphthalene and anthracene (Figures S9 and S10, Supporting Information), confirming its effectiveness in the structural‐specific identification of PAHs.^[^ ^22^, ^23^ ^]^

Based on the experimental results and a review of the literature, it was hypothesized that PAHs initially undergo a single electron transfer (ET) process. During oxidation, PAHs are believed to first generate stable radical cations (PAH⁺•) through single electron transfer.

(1) $$\mathrm{PAH}\to \mathrm{PA}H^{+}+e^{-}$$

Due to the strong electrophilicity of PAH⁺•, it readily reacts with nucleophilic substances in water, such as OH⁻ or H₂O, resulting in addition products. These addition products may also lose electrons again (further oxidation), leading to the formation of more stable carbonyl compounds. Therefore, it was hypothesized that the most common oxidation products of naphthalene are 1‐naphthol (A), 1,4‐dihydroxy‐1,4‐naphthoquinone (B), and 1,4‐naphthoquinone (C). The most common oxidation products of anthracene are 9‐anthrol (D), 9,10‐dihydroxy‐anthraquinone (E), and anthraquinone (F), which are likely generated through the addition of two oxygen atoms at the 9,10 positions of the anthracene molecule. This is probably due to the fact that the carbon atoms at positions 9 and 10 (located at the center of the central ring) in the generated PAH⁺• have the highest electron density and the lowest aromaticity. These two carbon atoms can disperse the electron cloud density of the free radical through conjugation with the adjacent rings, making them the most reactive sites for electrophilic addition reactions and significantly stabilizing the reaction intermediates. The most common oxidation products of pyrene are 1‐hydroxypyrene (G), 1,6‐dihydroxy‐pyrenone (H), 1,8‐dihydroxy‐pyrenone (I), 1,6‐pyrenone (J), and 1,8‐pyrenone (K). This is likely because the 1,6 and 1,8 positions of pyrene have higher electron density and weaker aromaticity, which facilitates the addition of water molecules at these positions. During subsequent oxidation, oxygen molecules can add to the 1,6 or 1,8 positions, forming carbonyl groups (Figure 5c).^[^ ^24^, ^25^, ^26^, ^27^, ^28^ ^]^

Based on the DPV test results (Figure 5d), fenitrothion exhibited a significant oxidation peak at +0.00 V (versus Ag/AgCl). When the optimized potential was applied for in situ EC‐SERS detection, its molecular skeletal vibration modes showed a marked enhancement at 942 cm⁻¹ (P‐O‐C symmetric stretching) and 1442 cm⁻¹ (C‐H in‐plane bending coupled with P═S vibration) (Figure 5e). Notably, these characteristic peaks were also observed to appear or disappear in the spectra of omethoate (Figure S11, Supporting Information), isocarbophos (Figure S12, Supporting Information), and profenofos (Figure S13, Supporting Information), indicating that the method exhibits structural‐specific response capabilities for organophosphorus pesticides.^[^ ^29^, ^30^ ^]^

The electrochemical oxidation‐reduction active sites of OPPs can be categorized into four main types. The first type involves the P═S bond present in the compound, which is more readily oxidized to P═O in the electrochemical environment. This oxidation leads to the formation of phosphoryl (P═O) compounds, significantly reducing the toxicity of the resulting phosphate derivatives. The second type corresponds to the reaction sites on the benzene ring, particularly those containing nitro (‐NO₂) groups. The nitro group is an electron‐withdrawing group, and in the electrochemical reaction, it significantly affects the electron density of the benzene ring, making the hydrogen atoms on the ring more prone to oxidation. During electrochemical reduction, the ‐NO₂ group itself is converted to ‐NH₂. The third type involves molecular structures that contain phosphorus (P) atoms and organic ester groups (such as ‐OR). During hydrolysis, the P─O─C bond reacts with water molecules, leading to bond cleavage. The products of this cleavage mainly include phosphate esters and alcohols. The fourth type involves the ‐Br group, which may be attributed to the polar covalent C‐Br bond in alkyl halides. Since Br has a higher electronegativity than C, the carbon atom carries a partial positive charge (δ+), while the bromine atom carries a partial negative charge (δ‐). Under an applied anodic potential in the electrochemical environment, the electron on the carbon atom (δ+) is more easily oxidized (lost). At the same time, the Br⁻ ion, due to its higher electron delocalization ability, stabilizes the negative charge and promotes the departure of the bromine ion (Figure 5f).^[^ ^10^, ^31^, ^32^, ^33^ ^]^

These results indicate that, under electrochemical oxidation, PAHs and OPPs generate oxidation‐state secondary molecular fingerprint information (Figure 5). The EC‐SERS detection method enhanced the molecular structural resolution capability of SERS, enabling the differentiation of similar PAH pollutants with highly consistent molecular structural units even under strong interference from extraction solvent molecules.

## 3 Conclusion

In conclusion, a self‐assembled 3D nanoporous Au microfluidic chip coupled with EC‐SERS was developed for the simultaneous detection of PAHs and OPPs. The chip's unique structure enhanced the sensitivity and reproducibility of SERS, achieving low LOD, good linearity, and high recovery in environmental samples. EC‐SERS also provided insights into the oxidation‐reduction processes of the pollutants. This research contributed to the development of advanced analytical platforms for environmental monitoring and pollution control. The system was shown to hold significant promise for a wide range of applications, including environmental safety, water quality monitoring, and public health protection, paving the way for more effective and accessible detection methods.

## 4 Experimental Section

### Materials and Reagents

Benchmark samples of anthracene, pyrene, naphthalene, and ascorbic acid were purchased from Shanghai Aladdin Biochemical Technology Co., Ltd. Benchmark samples of omethoate, profenofos, fenitrothion, and isocarbophos were obtained from Beijing Honghu United Chemical Products Co., Ltd. PDMS prepolymer was supplied by Beijing Yancheng Technology Co., Ltd. All chemicals used in the experiments were of analytical grade and were applied without further purification.

### Preparation of the 3D Au/Ag NPs Substrate

The preparation of 3D Au/Ag NPs was carried out analogous to the procedure reported in the literature.^[^ ^19^ ^]^ Briefly, quartz substrates were pretreated by repeatedly rinsing with ethanol and distilled water, followed by plasma cleaning of the quartz substrates for 30 min. Magnetron sputtering was then employed to deposit a trilayer structure (20 nm Au / 140 nm Ag / 20 nm Au) onto the pretreated quartz substrates patterned with a working electrode.^[^ ^34^ ^]^ The samples were alloyed at 500 °C in Ar atmosphere for 5 min, and subsequently immersed in 65% HNO₃ for 5 min to obtain Au NPs with a nanoporous structure. Then, Ag nanoclusters were controllably loaded onto the surface of the 3D‐Au substrate via a wet cyclic deposition method. Specifically, the pretreated 3D‐Au substrate was alternately immersed in a 5 mM silver nitrate (AgNO₃) precursor solution and a 10 mM ascorbic acid reducing agent solution. Each deposition cycle consisted of a 30 min adsorption step in the precursor solution followed by an in situ reduction process. The loading density of Ag nanoclusters was tuned by varying the number of deposition cycles. The 3D‐Au substrates with varying numbers of Ag deposition cycles (XC) were denoted as 3D‐Au‐Ag‐XC, where XC refers to the number of wet cyclic deposition steps applied for Ag nanoparticle loading. During the process, deionized water was used to rinse the substrate between cycles to eliminate residual ions at the interface. The resulting 3D‐Au‐Ag substrates were finally purged with nitrogen and stored in a vacuum drying oven at 50 °C (Figure 1a).

### Fabrication of 3D Au/Ag NPs SERS Chips

The overall chip was prepared by bonding a quartz substrate at the bottom with a PDMS substrate on the top. The bottom substrate was fabricated using standard photolithography and wet etching to form the electrolyte cell and electrode channels, while the top substrate was prepared using standard photolithography to create the electrolyte cell channels, and the substrate preparation was completed. Then, Au/Ag NPs were deposited onto the etched chips through magnetron sputtering, as described in Section 4.2. The top PDMS substrate was fabricated in three steps. First, a mold was created by washing a silicon wafer three times with acetone and isopropanol, followed by drying and applying a silicon wafer modifier. After 15 min, a layer of negative photoresist was evenly applied on the wafer, baked, exposed, and developed. In the second step, the prepared silicon template was treated with 3–5 drops of trimethylchlorosilane at 60 °C for 15 min, then cooled to room temperature. PDMS was prepared by weighing 40 g of PDMS and adding 4 g of curing agent, followed by stirring until the mixture turned milky white. The mixture was degassed in a vacuum pump and poured into the mold, then cured at 80 °C for 4 h. Finally, two circular channels with a diameter of 0.2 cm were created on the prepared PDMS cover. The fabricated quartz substrate and PDMS cover plate were surface‐activated by oxygen plasma treatment and subsequently conformally bonded to form an enclosed microchamber structure. The platinum wire counter electrode and Ag/AgCl reference electrode were then precisely inserted into the predesigned electrode slots within the microchamber. Finally, capillary tubes (I.D = 200 µm, O.D = 360 µm) were integrated at the upper end of the chip using epoxy resin encapsulation (Figure 1b).

### Sample Preparation for SERS and EC‐SERS Measurement

To prepare the PAHs standard solution, the solid samples were first accurately weighed, followed by dissolving in an appropriate amount of acetonitrile. The solution was then transferred into a volumetric flask and diluted to the desired volume, resulting in a 1 g L^−1^ stock solution of anthracene, naphthalene, and pyrene, which was stored in a refrigerator at 4 °C. The preparation of the OPPs standard solution followed the same procedure as that of the PAHs solution. The molecular structures of the three PAHs and four OPPs analyzed in this experiment are illustrated in Figure S1 (Supporting Information).

The LiClO_4_ solid sample was accurately weighed and dissolved in ultrapure water to prepare a 0.1 mol L^−1^ LiClO_4_ aqueous solution, which was used as the electrolyte for PAHs detection. Na_2_HPO_4_ and C_6_H_8_O_7_ solid samples were accurately weighed and dissolved separately in ultrapure water, with the pH adjusted to neutral. The resulting Na_2_HPO_4_‐C_6_H_8_O_7_ aqueous solution was used as the electrolyte for OPPs detection.

### Qualitative of the Quantitative SERS Detection of PAHs and OPPs

Different experimental methods, both dry and wet processes, were applied to test three PAHs (anthracene, pyrene, and naphthalene) and four OPPs (fenitrothion, profenofos, omethoate, and isocarbophos). In the dry process, a measured amount of the solution was dropped onto the substrate surface and dried before conducting SERS detection. In the wet process, the solution was directly dropped onto the substrate without drying, allowing the liquid to cover the substrate for immediate testing. A 785 nm laser, L50X objective lens, and CCD detector were used. During the dry testing, the laser power was set at 0.1%, 0.1%, 0.5%, 1%, 5%, 1%, and 5% for the respective substances, while for the wet testing, the power was 10% for PAHs and 50% for OPPs. The integration time was 10 s, and five spectra were collected for each sample. The averaged data were then processed by background subtraction to obtain the final Raman spectra.

### Method Verification and Real Sample Analysis

SERS detection of seven samples was performed using 3D Au‐Ag NPs, with the detection limits and linear ranges determined for each. A qualitative and quantitative SERS analysis method for environmental water samples was established.

Water samples were collected from different locations in Baiyangdian and labeled as Sample 1, 2, and 3 in subsequent experiments. The water samples were stored in brown glass bottles at 4 °C and analyzed within 24 h. Before analysis, the environmental water samples were filtered using a 0.22 µm membrane.

### In‐Situ Raman Measurement of PAHs and OPPs

After the chip preparation was completed, it was connected to an electrochemical workstation. Ag/AgCl was used as the reference electrode, 3D Au‐Ag NPs as the working electrode, and a platinum wire as the counter electrode. A potentiostat was employed for subsequent ECD‐SERS tests. The prepared standard solutions were mixed with the electrolyte and introduced into the microchip. To evaluate the ECD performance of the three‐electrode system in the microchip, linear sweep voltammetry (LSV) tests were conducted on 0.1 mol L^−1^ KCl solutions containing different concentrations of K_3_[Fe(CN)_6_]/K_4_[Fe(CN)_6_].

## Conflict of Interest

The authors declare no conflicts of interest.

## Supporting information



Supporting Information

## Data Availability

The data that support the findings of this study are available from the corresponding author upon reasonable request.

## References

[1] C. Chen , G. Liu , C. Zhao , M. Wang , Y. Yang , L. Yang , M. Zheng , J. Hazard. Mater. 2025, 488, 137270. 39864202 10.1016/j.jhazmat.2025.137270

[2] X. Cheng , L. Gao , X. Cao , Y. Zhang , Q. Ai , J. Weng , Y. Liu , J. Li , L. Zhang , B. Lyu , Y. Wu , M. Zheng , Environ. Sci. Technol. 2024, 58, 11935. 38913859 10.1021/acs.est.4c02909

[3] Z. Chen , M. Cai , H. Zheng , Y. Gao , Y. Xia , J. Hazard. Mater. 2025, 481, 136528. 39556912 10.1016/j.jhazmat.2024.136528

[4] C. Liu , X. Cao , X. Ma , Y. Wang , M. Zhang , J. Qiu , J. Chen , H. Xue , Small 2025, 21, 2411212. 10.1002/smll.202411212 39981875

[5] J. Zhou , D. Xiong , H. Zhang , J. Xiao , R. Huang , Z. Qiao , Z. Yang , Z. Zhang , Environ. Sci. & Technol. 2025, 59, 1844. 39813103 10.1021/acs.est.4c13849

[6] X. Xu , T. Li , Y. Liu , Y. Xu , J. Zhao , W. Chen , Y. Luo , L. Han , W. Song , R. Yang , P. He , Y. Wang , H. Zhou , Adv. Funct. Mater. 2024, 34, 2407336.

[7] V. J. Esposito , R. C. Fortenberry , C. Boersma , L. J. Allamandola , J. Phys. Chem. A 2025, 129, 244. 39714277 10.1021/acs.jpca.4c07416

[8] J. Khoubi , A. Ghiasvand , A. Bahrami , F. G. Shahna , M. Farhadian , Microchem. J. 2024, 199, 110088. 10.1016/j.chroma.2024.465387 39326383

[9] N. H. Sazali , M. Miskam , F. B. M. Suah , N. Y. Rahim , Korean J. Chem. Eng. 2024, 41, 1725.

[10] J. Chen , D. Dong , S. Ye , RSC Adv. 2018, 8, 4726. 35539546 10.1039/c7ra11927e PMC9077747

[11] A. Garcia Cruz , I. Haq , T. Cowen , S. Di Masi , S. Trivedi , K. Alanazi , E. Piletska , A. Mujahid , S. A. Piletsky , Biosens. Bioelectron. 2020, 169, 112536. 32980804 10.1016/j.bios.2020.112536

[12] M. Chen , J. Zhang , X. Zhu , Z. Liu , J. Huang , X. Jiang , F. Fu , Z. Lin , Y. Dong , ACS Appl. Mater. Interfaces 2022, 14, 26216. 35605108 10.1021/acsami.2c04087

[13] Y. Yan , H. Liu , C. Liu , Y. Zhao , S. Liu , D. Wang , M. Fritz , A. Ispas , A. Bund , P. Schaaf , X. Wang , Appl. Mater. Today 2021, 25, 101185.

[14] N. Sun , B. Huang , Z. Lv , N. Ran , Y. Gan , J. Zhang , Anal. Chem. 2024, 96, 9104. 38775358 10.1021/acs.analchem.4c00657

[15] J.‐C. Dong , X.‐G. Zhang , V. Briega‐Martos , X. Jin , J. Yang , S. Chen , Z.‐L. Yang , D.‐Y. Wu , J. M. Feliu , C. T. Williams , Z.‐Q. Tian , J.‐F. Li , Nat. Energy 2018, 4, 60.

[16] L. Long , W. Ju , H. Y. Yang , Z. Li , ACS Mater. Au 2022, 2, 552. 36855623 10.1021/acsmaterialsau.2c00005 PMC9928417

[17] M. L. Pedano , S. Li , G. C. Schatz , C. A. Mirkin , Angew. Chem. Int. Ed. Engl. 2010, 49, 78. 19967681 10.1002/anie.200904646

[18] D. Wang , P. Schaaf , J. Mater. Chem. 2012, 22.

[19] Y. Yan , A. I. Radu , W. Rao , H. Wang , G. Chen , K. Weber , D. Wang , D. Cialla‐May , J. Popp , P. Schaaf , Chem. Mater. 2016, 28, 7673.

[20] M. Li , H. Yu , Y. Cheng , Y. Guo , W. Yao , Y. Xie , Ecotoxicol. Environ. Saf. 2020, 200, 110780. 32470683 10.1016/j.ecoenv.2020.110780

[21] E. Mathieu‐Scheers , S. Bouden , C. Grillot , J. Nicolle , F. Warmont , V. Bertagna , B. Cagnon , C. Vautrin‐Ul , J. Electroanal. Chem. 2019, 848, 113253.

[22] E. M. Espinoza , J. A. Clark , J. B. Derr , D. Bao , B. Georgieva , F. H. Quina , V. I. Vullev , ACS Omega 2018, 3, 12857. 31458010 10.1021/acsomega.8b01581 PMC6644773

[23] S. Karakaya , İ. Kaya , Polymer 2021, 212, 123300.

[24] R. Fan , H. Tian , Q. Wu , Y. Yi , X. Yan , B. Liu , J. Hazard. Mater. 2022, 422, 126959. 34449353 10.1016/j.jhazmat.2021.126959

[25] T. M. Figueira‐Duarte , K. Mullen , Chem. Rev. 2011, 111, 7260. 21740071 10.1021/cr100428a

[26] L. Ji , I. Krummenacher , A. Friedrich , A. Lorbach , M. Haehnel , K. Edkins , H. Braunschweig , T. B. Marder , J. Org. Chem. 2018, 83, 3599. 29480011 10.1021/acs.joc.7b03227

[27] J. Merz , J. Fink , A. Friedrich , I. Krummenacher , H. H. Al Mamari , S. Lorenzen , M. Haehnel , A. Eichhorn , M. Moos , M. Holzapfel , H. Braunschweig , C. Lambert , A. Steffen , L. Ji , T. B. Marder , Chem. 2017, 23, 13164. 10.1002/chem.201702594 28718975

[28] K. Müllen , Helv. Chim. Acta 2004, 61, 2307.

[29] T. Yaseen , D.‐W. Sun , H. Pu , T.‐T. Pan , Food Analytical Methods 2018, 11, 2518.

[30] A. Zengin , U. Tamer , T. Caykara , J. Raman Spectrosc. 2017, 49, 452.

[31] L. Fang , L. Xu , N. Zhang , Q. Shi , T. Shi , X. Ma , X. Wu , Q. X. Li , R. Hua , J. Hazard. Mater. 2021, 417, 126024. 33992014 10.1016/j.jhazmat.2021.126024

[32] B. Gao , S. Zhao , Z. Zhang , L. Li , K. Hu , A. E. Kaziem , Z. He , X. Hua , H. Shi , M. Wang , Environ. Int. 2019, 127, 694. 30991225 10.1016/j.envint.2019.04.018

[33] H. J. Kim , C. J. Lee , M. R. Karim , M. S. Kim , M. S. Lee , Spectrochimica Acta Part A – Molecul. Biomol. Spectr. 2011, 78, 179. 10.1016/j.saa.2010.09.018 20965774

[34] X. Wang , Z. Guo , D. Zhang , Y. Yan , Y. Yu , B. Du , Z. Zhang , X. Wang , Sens. Actuators, B 2024, 407.

