# Supplementary material for: A Miniaturized Electrochemical SERS Chip with 3D Nanoporous Gold Interface for Qualitative and Quantitative Analysis of Organic Pollutants and their Oxidation Intermediates
Source: Small. 2025 Jul 23;21(37):e03894. doi: 10.1002/smll.202503894 (PMC12444819; doi:10.1002/smll.202503894)
Supplement: Supplementary file 1 — Supporting Information [file SMLL-21-e03894-s001.docx]

Supporting Information

**A Miniaturized Electrochemical SERS Chip with 3D Nanoporous Gold Interface for Qualitative and Quantitative Analysis of Organic Pollutants and Their Oxidation Intermediates**

*Xu Wang,^1,2^ Yong Yan,^1^* Huan Zhou,^1^ Xinyu Liu,^1^ Riyi Zhang,^1^ Dong Wang,^3^* Peter Schaaf,**^3^ Guangsheng Guo^1^, Xiayan Wang^1^**

**Table of Contents**

[**Figure S1.** (a) Chemical Structures of PAHs and (b) Chemical Structures of OPPs.](#_Toc202882198) (**Page 4**)

[**Figure S2.** SEM images of 3D Au NPs obtained from the alloying temperature of 400 °C (a) and 700 °C (b); Statistical analysis of the average width of Au nanoribbons in 3D Au NPs prepared under different alloying annealing temperatures: 450°C (c), 500°C (d), 550°C (e), and 600°C (f).](#_Toc202882199) (**Page 5**)

[**Figure S3.** Electrochemical characterization of the 3D Au-Ag NPs Microchip: LSV results of K_3_[Fe(CN)_6_]/K_4_[Fe(CN)_6_] redox system (a); Standard curve (b).](#_Toc202882200) (**Page 6**)

[**Figure S4.** Standard curve of omethoate within the ppb concentration range (a) and stability (b).](#_Toc202882201) (**Page 6**)

[**Figure S5.** Standard curve of isocarbophos within the ppb concentration range (a) and stability (b).](#_Toc202882202) (**Page 7**)

[**Figure S6.** Standard curve of profenofos within the ppb concentration range (a) and stability (b).](#_Toc202882203) (**Page 7**)

[**Figure S7.** Standard curve of naphthalene within the ppb concentration range (a) and stability (b).](#_Toc202882204) (**Page 8**)

[**Figure S8.** Standard curve of anthracene within the ppb concentration range (a) and stability (b).](#_Toc202882205) (**Page 8**)

[**Figure S9.** Selective analysis of 50 ppb PAHs-naphthalene using the Microchip EC-SERS detection method at applied electrochemical potentials: (a) DPV curve of naphthalene; (b) Raman spectrum of naphthalene; (c) proposed oxidation mechanism of naphthalene.](#_Toc202882206) (**Page 9**)

[**Figure S10.** Selective analysis of 50 ppb PAHs-anthracene using the Microchip EC-SERS detection method at applied electrochemical potentials: (d) DPV curve of anthracene; (e) Raman spectrum of anthracene; (f) proposed oxidation mechanism of anthracene.](#_Toc202882207) (**Page 9**)

[**Figure S11.** Selective analysis of 50 ppb OPPs-omethoate using the Microchip EC-SERS detection method at applied electrochemical potentials: (d) DPV curve of omethoate; (e) Raman spectrum of omethoate; (f) proposed oxidation mechanism of omethoate.](#_Toc202882208) (**Page 10**)

[**Figure S12.** Selective analysis of 50 ppb OPPs-isocarbophos using the Microchip EC-SERS detection method at applied electrochemical potentials: (g) DPV curve of isocarbophos; (h) Raman spectrum of isocarbophos; (i) proposed oxidation mechanism of isocarbophos.](#_Toc202882209) (**Page 10**)

[**Figure S13.** Selective analysis of 50 ppb OPPs-profenofos using the Microchip EC-SERS detection method at applied electrochemical potentials: (j) DPV curve of profenofos; (k) Raman spectrum of profenofos; (l) proposed oxidation mechanism of profenofos.](#_Toc202882210) (**Page 11**)

[**Figure S14.** HR-TEM images of the 3D-Au-Ag substrate. (a) Morphology of the nanoporous framework; (b) Dark-field contrast indicating Ag nanoclusters (highlighted by red arrows); (c) Lattice fringes showing interplanar spacings; (d, e) Additional images of Ag nanoclusters with darker contrast (highlighted by red arrows); (f) High-resolution lattice fringes corresponding to Au (111), Au (200), and Ag (220) planes.](#_Toc202882212) (**Page 11-12**)

[**Figure S15.** Localized electric field distribution and plasmonic hotspot mapping of the 3D-Au substrate under incident light at different wavelengths, as simulated by FDTD analysis.](#_Toc202882214) (**Page 12**)

[**Figure S16.** The stability result of pyrene and fenitrothion (50 ppm).](#_Toc202882216) (**Page 12**)

[**Table S1.** Analytical performance of SERS for PAHs and OPPs.](#_Toc202882217) (**Page 13**)


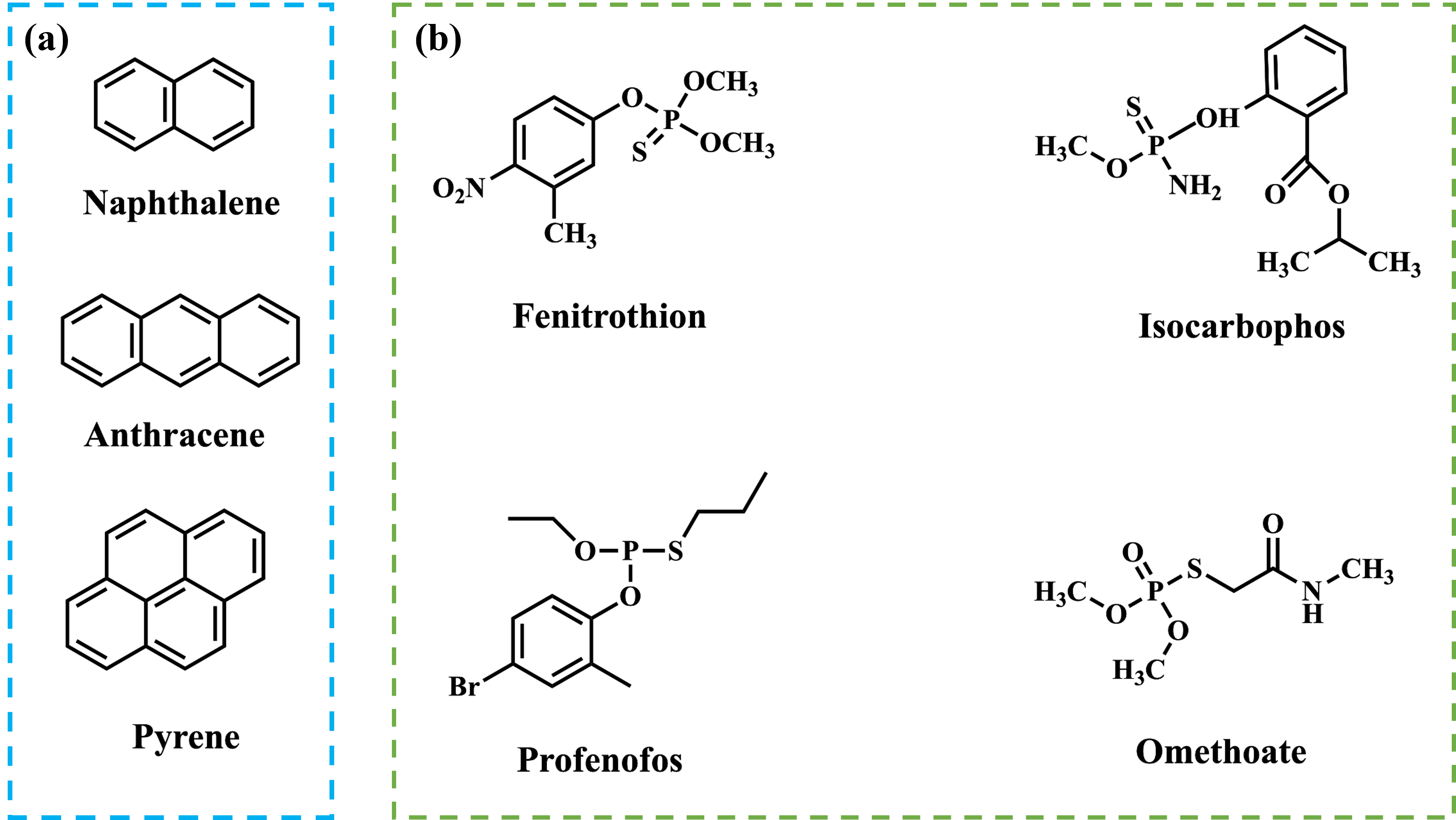


**Figure S1.** (a) Chemical Structures of PAHs and (b) Chemical Structures of OPPs.


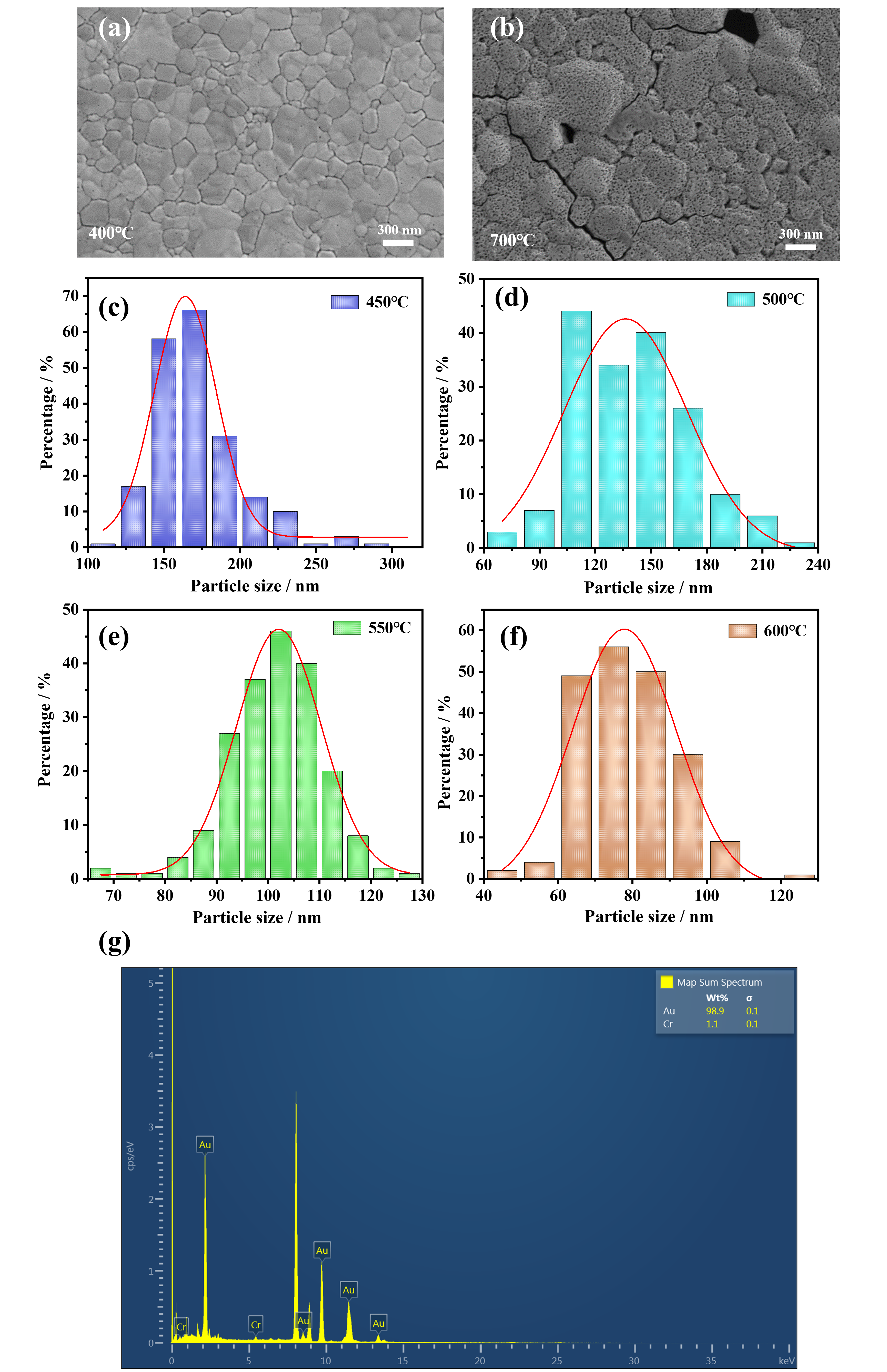


**Figure S2.** SEM images of 3D Au NPs obtained from the alloying temperature of 400 °C (a) and 700 °C (b); Statistical analysis of the average width of Au nanoribbons in 3D Au NPs prepared under different alloying annealing temperatures: 450°C (c), 500°C (d), 550°C (e), and 600°C (f).


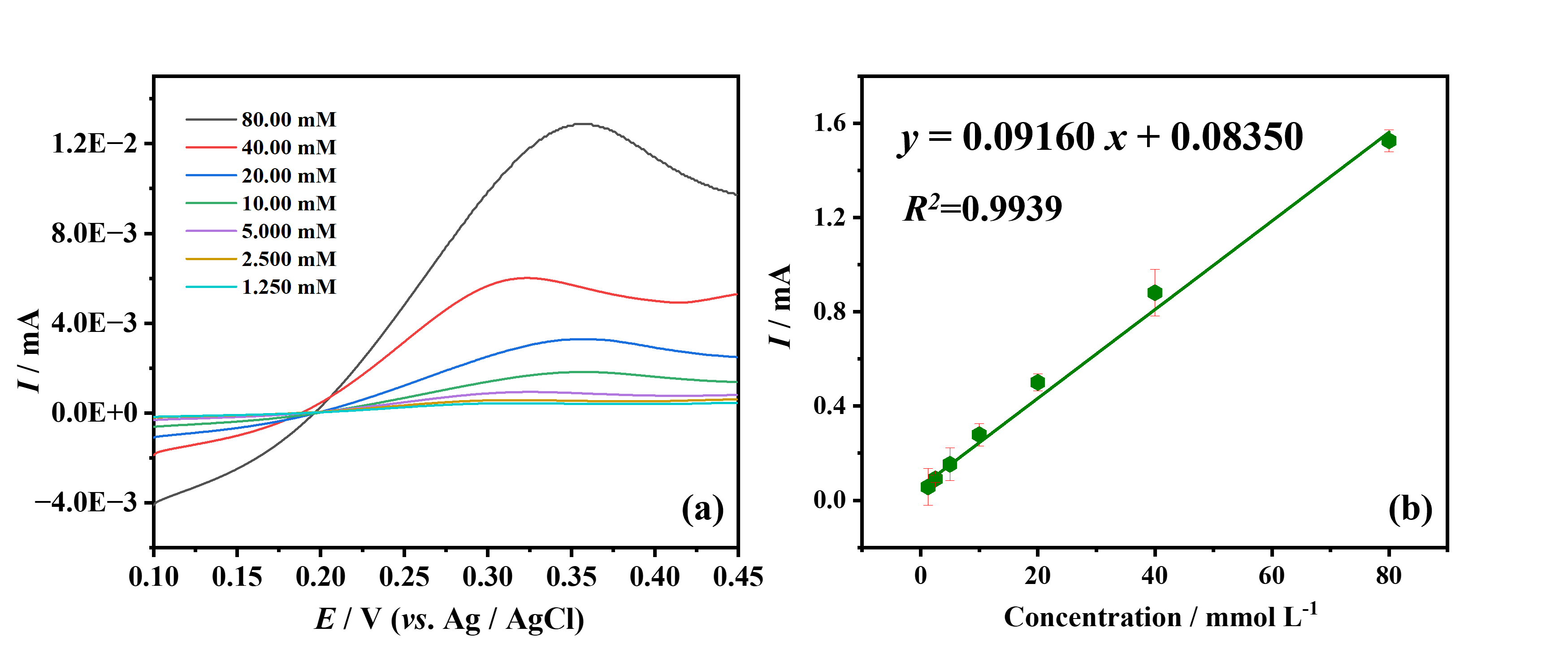


**Figure S3.** Electrochemical characterization of the 3D Au-Ag NPs Microchip: LSV results of K_3_[Fe(CN)_6_]/K_4_[Fe(CN)_6_] redox system (a); Standard curve (b).


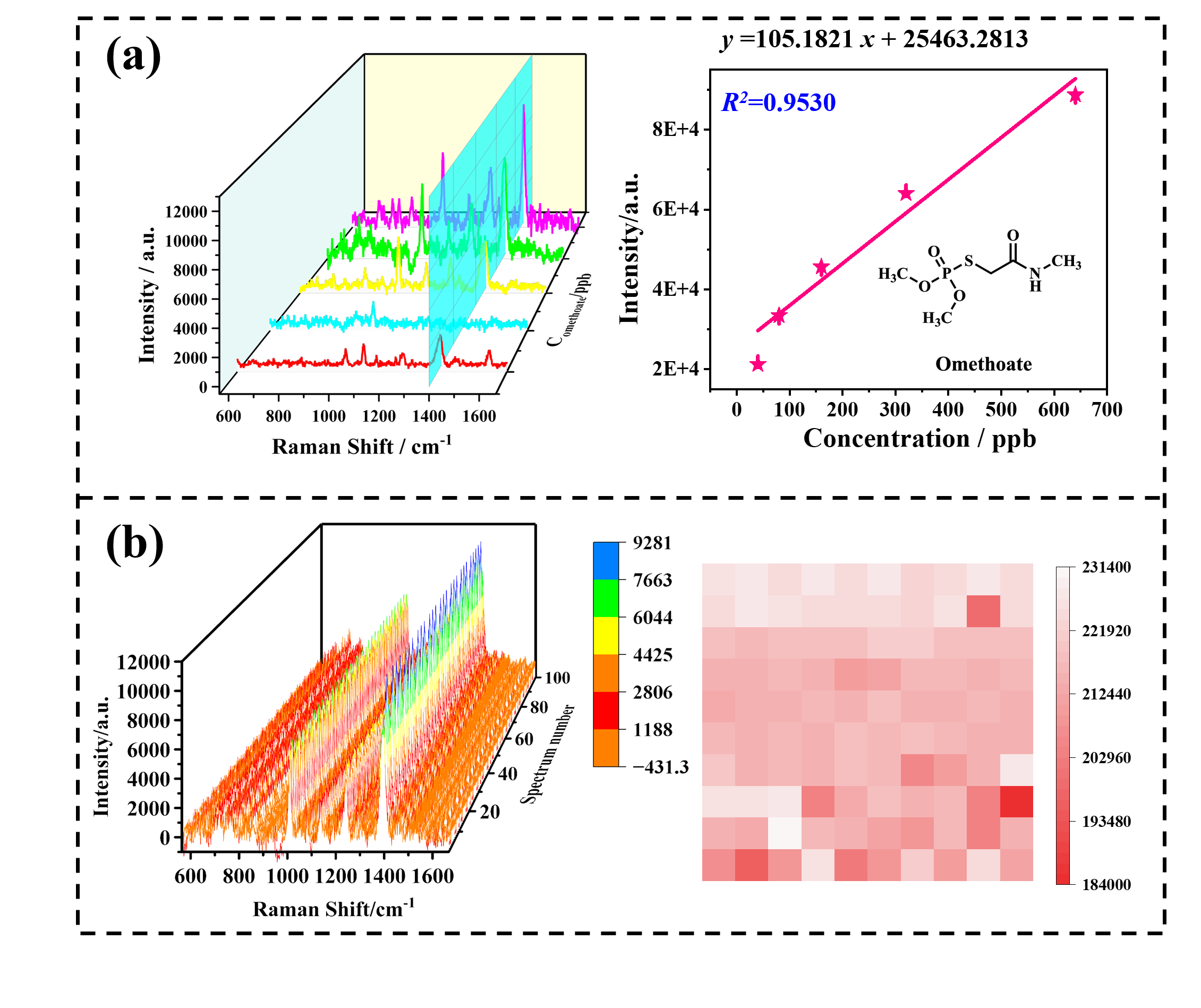


**Figure S4.** Standard curve of omethoate within the ppb concentration range (a) and stability (b).


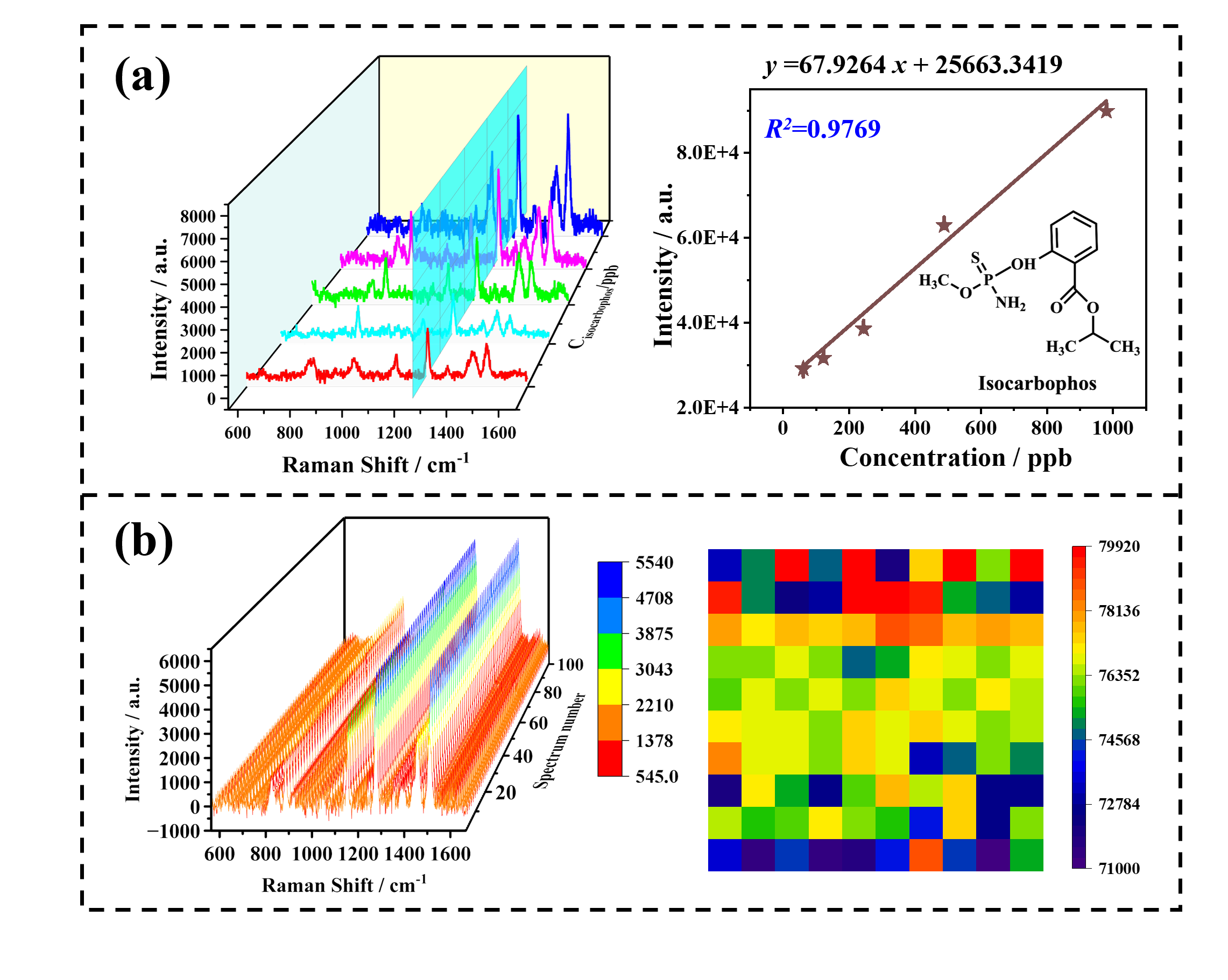


**Figure S5.** Standard curve of isocarbophos within the ppb concentration range (a) and stability (b).


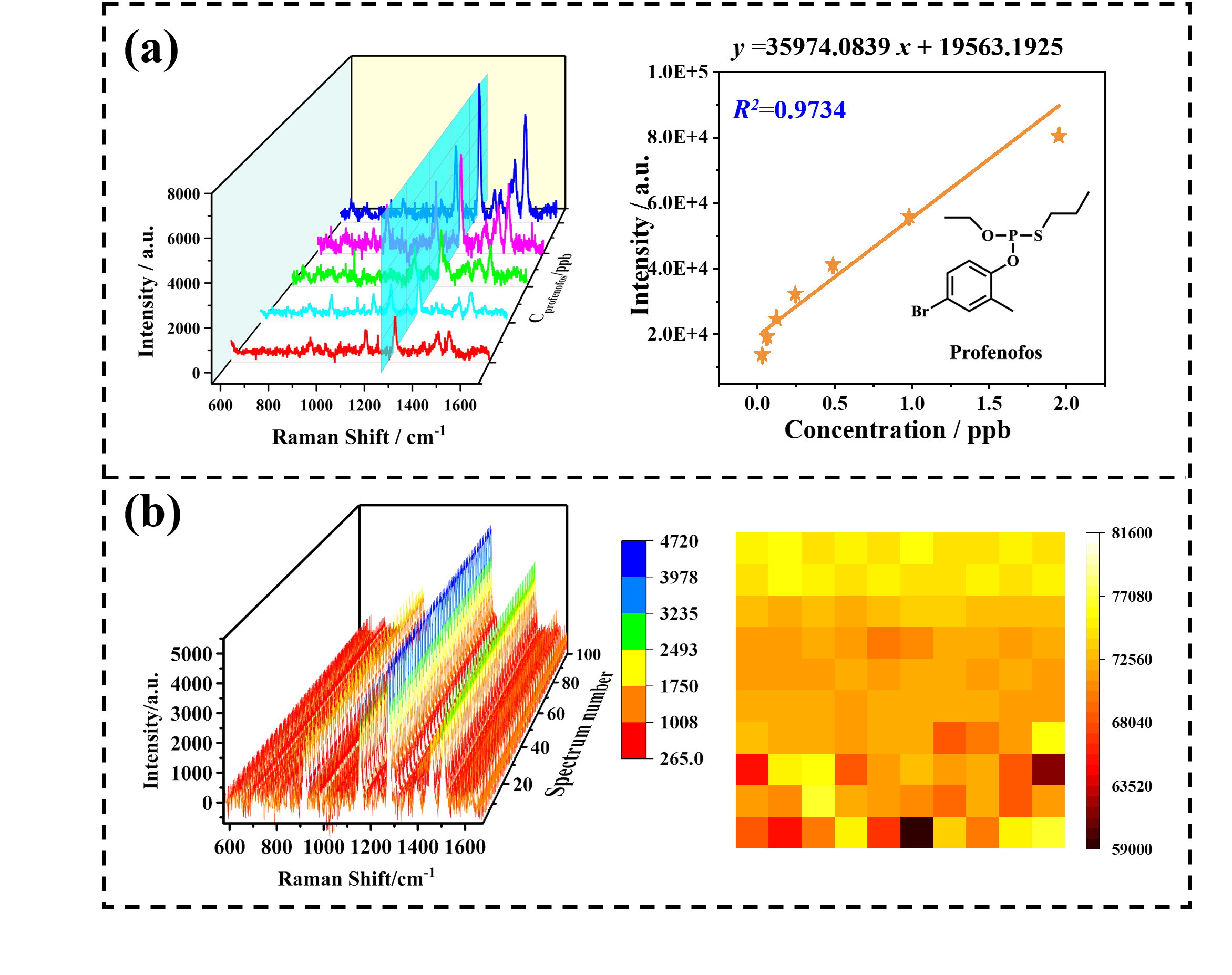


**Figure S6.** Standard curve of profenofos within the ppb concentration range (a) and stability (b).


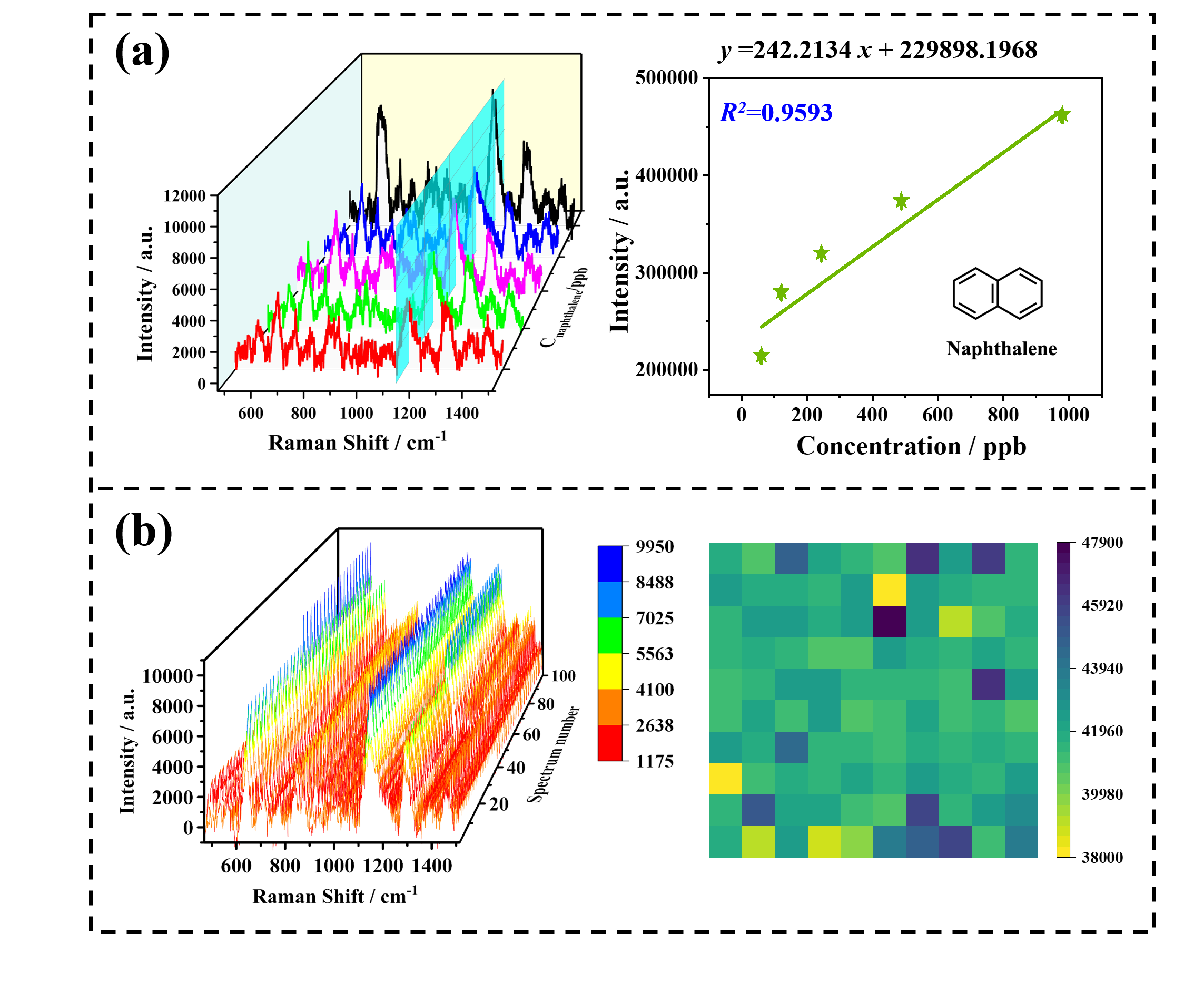


**Figure S7.** Standard curve of naphthalene within the ppb concentration range (a) and stability (b).


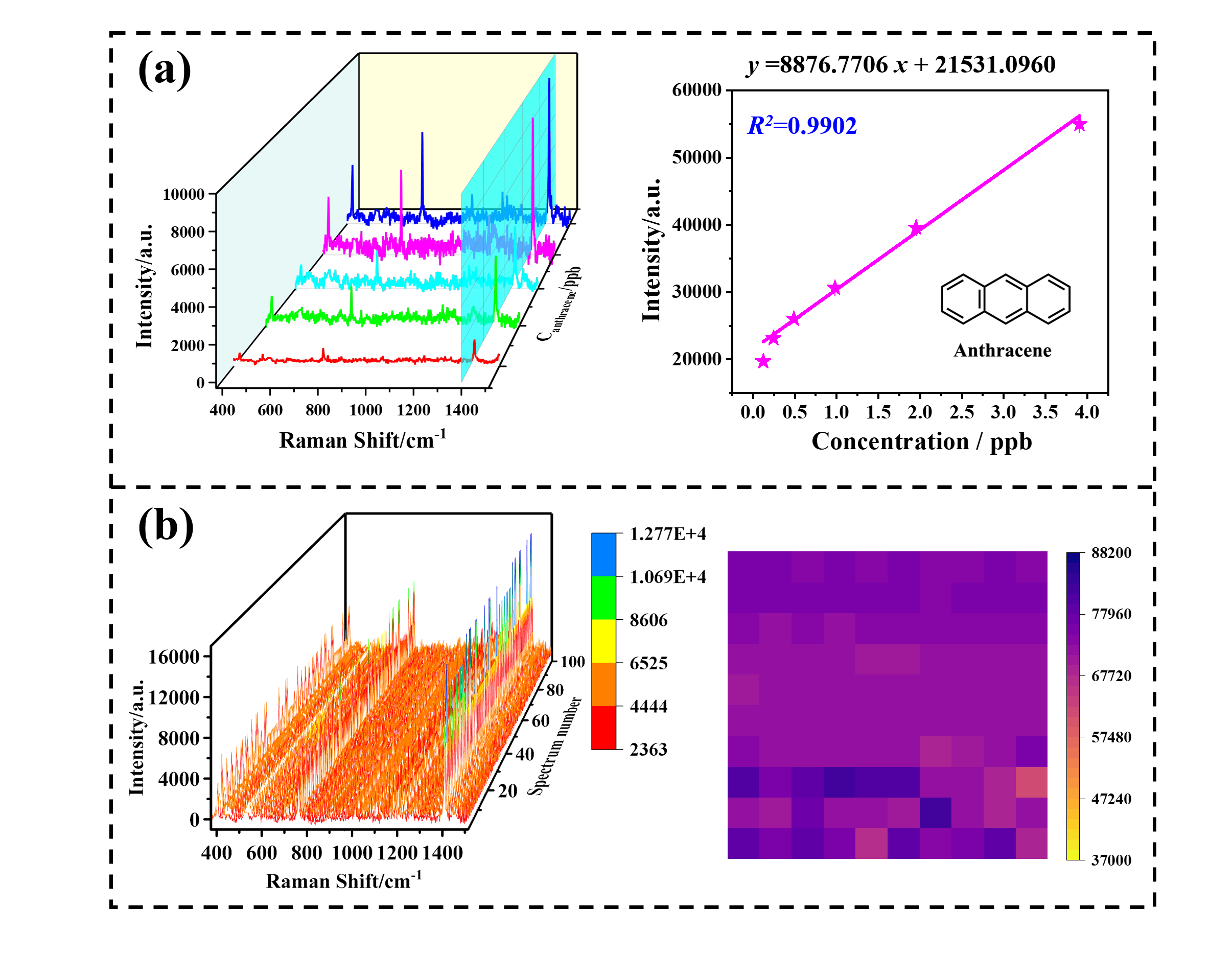


**Figure S8.** Standard curve of anthracene within the ppb concentration range (a) and stability (b).


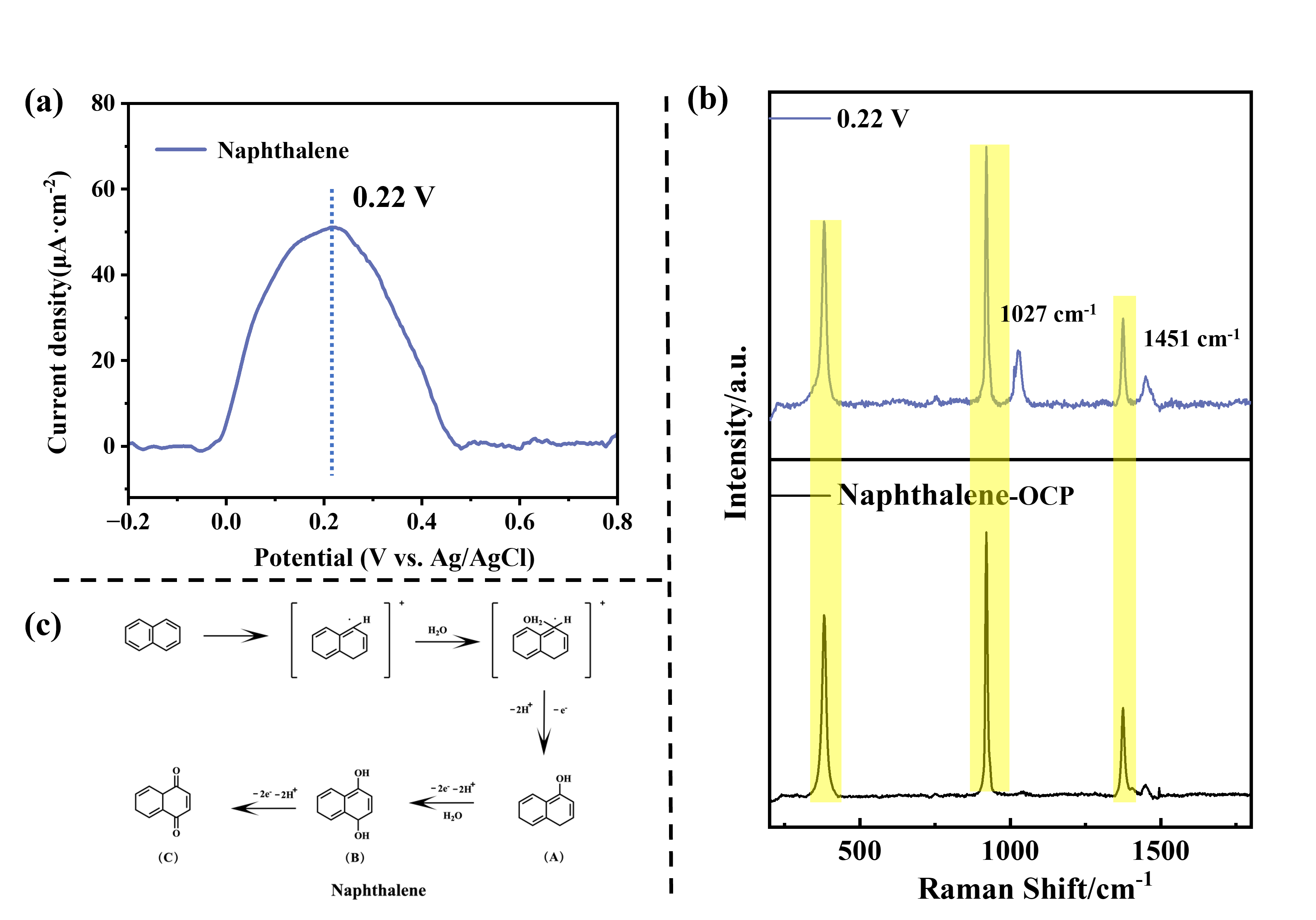


**Figure S9.** Selective analysis of 50 ppb PAHs-naphthalene using the Microchip EC-SERS detection method at applied electrochemical potentials: (a) DPV curve of naphthalene; (b) Raman spectrum of naphthalene; (c) proposed oxidation mechanism of naphthalene.


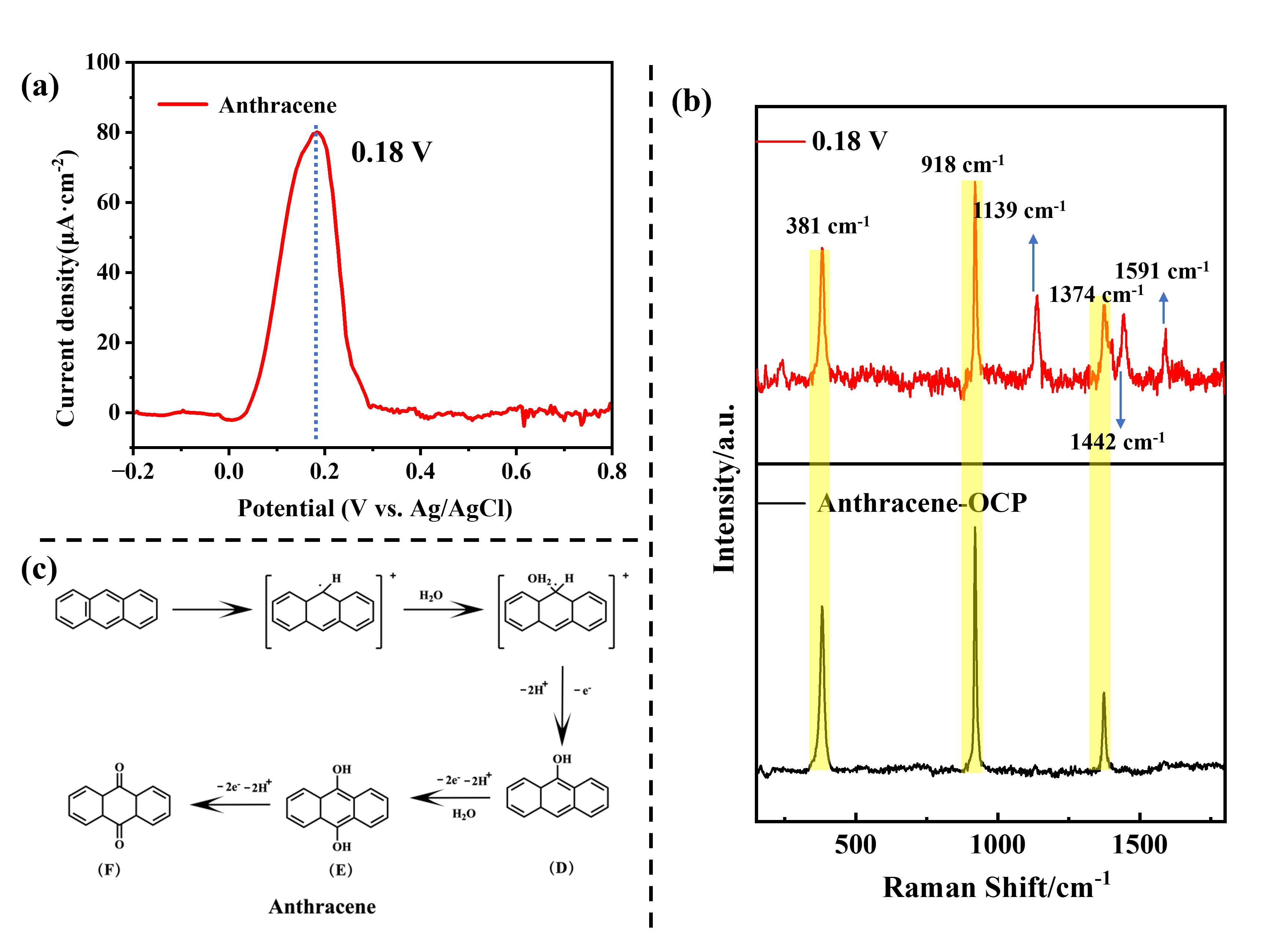


**Figure S10.** Selective analysis of 50 ppb PAHs-anthracene using the Microchip EC-SERS detection method at applied electrochemical potentials: (d) DPV curve of anthracene; (e) Raman spectrum of anthracene; (f) proposed oxidation mechanism of anthracene.


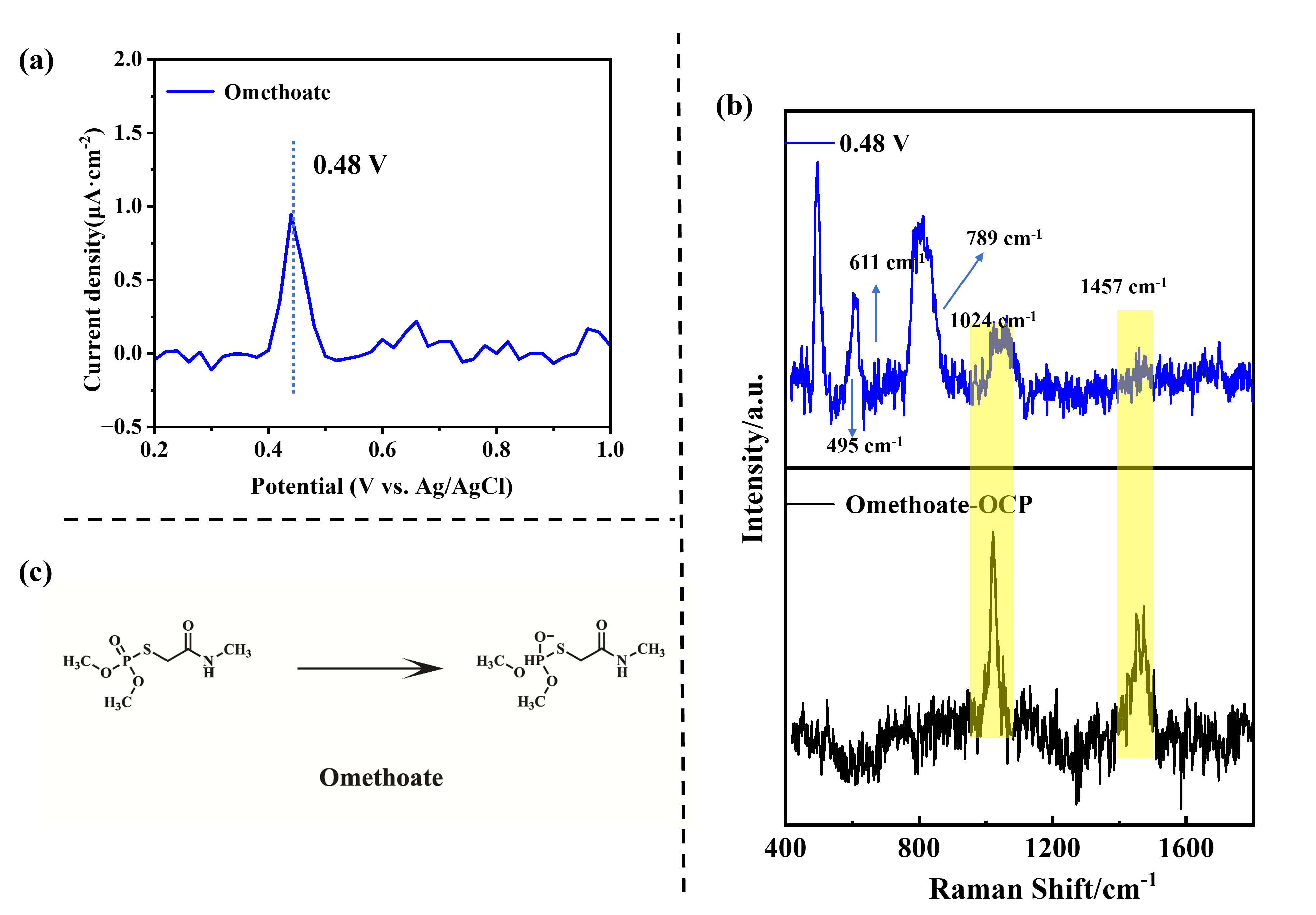


**Figure S11.** Selective analysis of 50 ppb OPPs-omethoate using the Microchip EC-SERS detection method at applied electrochemical potentials: (d) DPV curve of omethoate; (e) Raman spectrum of omethoate; (f) proposed oxidation mechanism of omethoate.


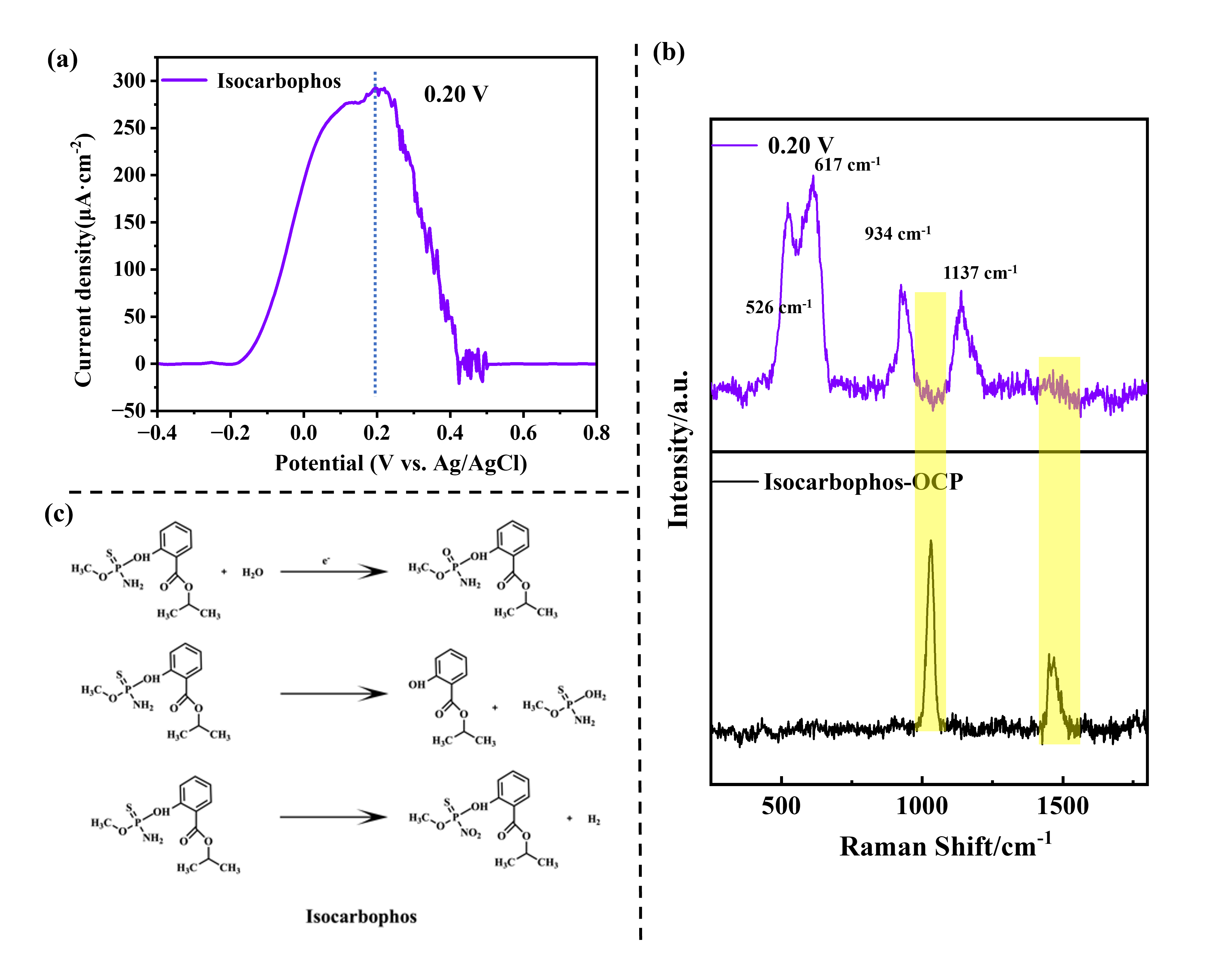


**Figure S12.** Selective analysis of 50 ppb OPPs-isocarbophos using the Microchip EC-SERS detection method at applied electrochemical potentials: (g) DPV curve of isocarbophos; (h) Raman spectrum of isocarbophos; (i) proposed oxidation mechanism of isocarbophos.


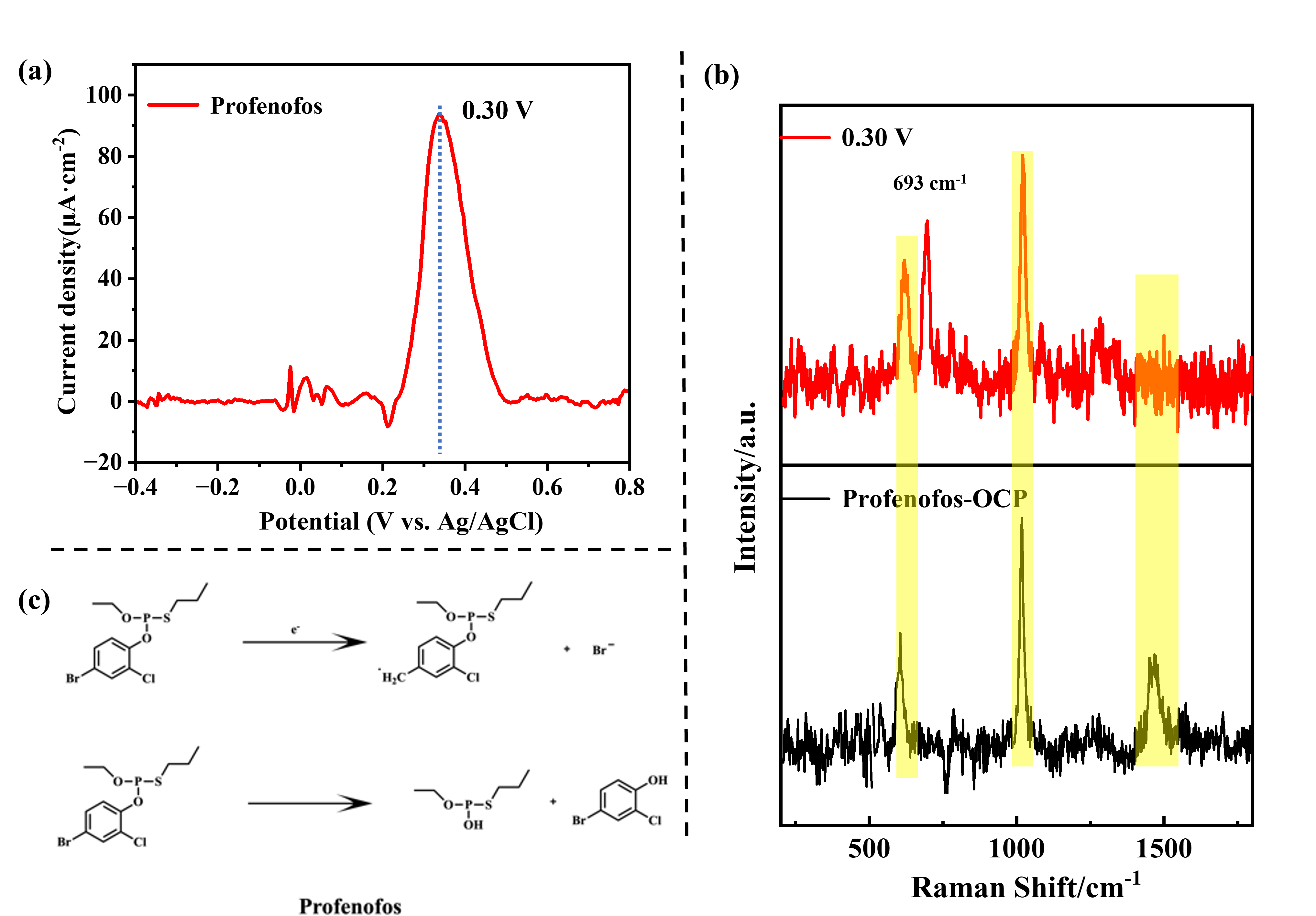


**Figure S13.** Selective analysis of 50 ppb OPPs-profenofos using the Microchip EC-SERS detection method at applied electrochemical potentials: (j) DPV curve of profenofos; (k) Raman spectrum of profenofos; (l) proposed oxidation mechanism of profenofos.


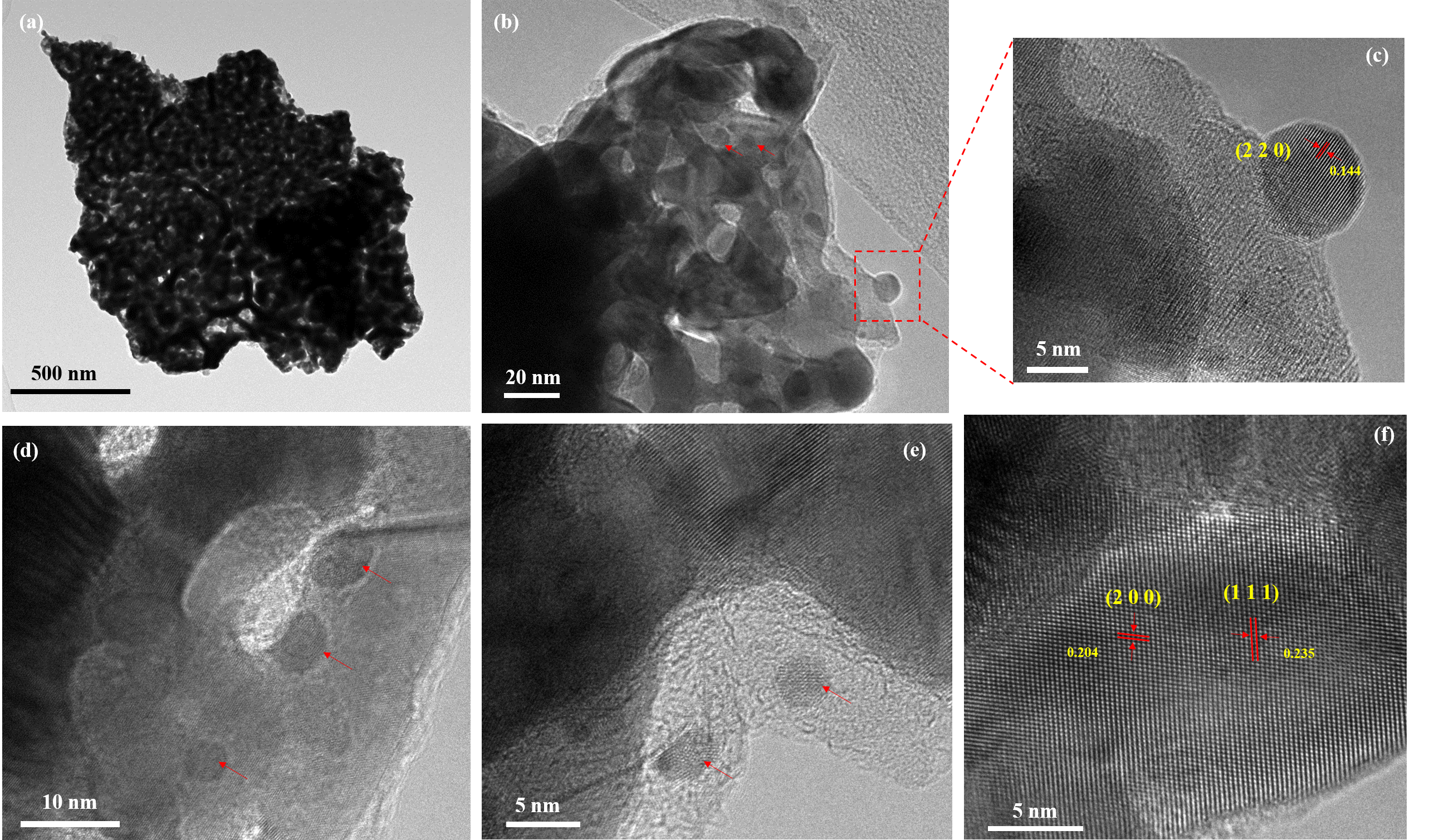


**Figure S14.** HR-TEM images of the 3D-Au-Ag substrate. (a) Morphology of the nanoporous framework; (b) Dark-field contrast indicating Ag nanoclusters (highlighted by red arrows); (c) Lattice fringes showing interplanar spacings; (d, e) Additional images of Ag nanoclusters with darker contrast (highlighted by red arrows); (f) High-resolution lattice fringes corresponding to Au (111), Au (200), and Ag (220) planes.


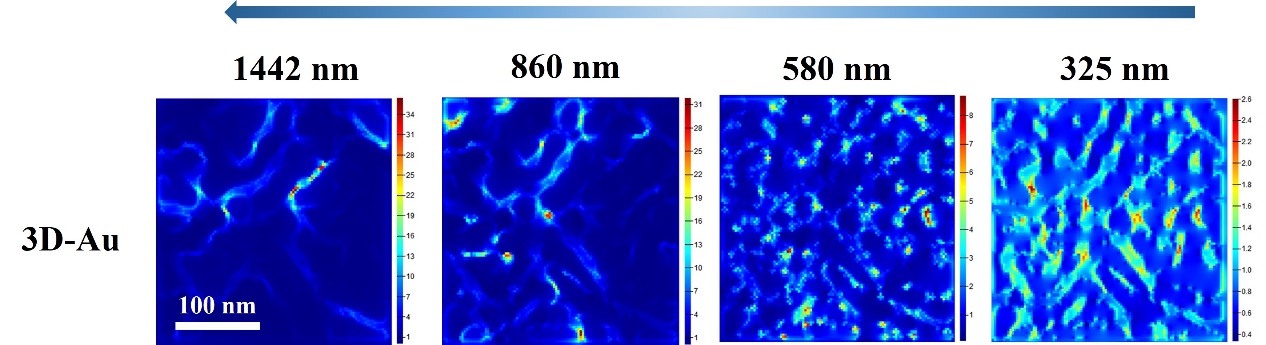


**Figure S15.** Localized electric field distribution and plasmonic hotspot mapping of the 3D-Au substrate under incident light at different wavelengths, as simulated by FDTD analysis.


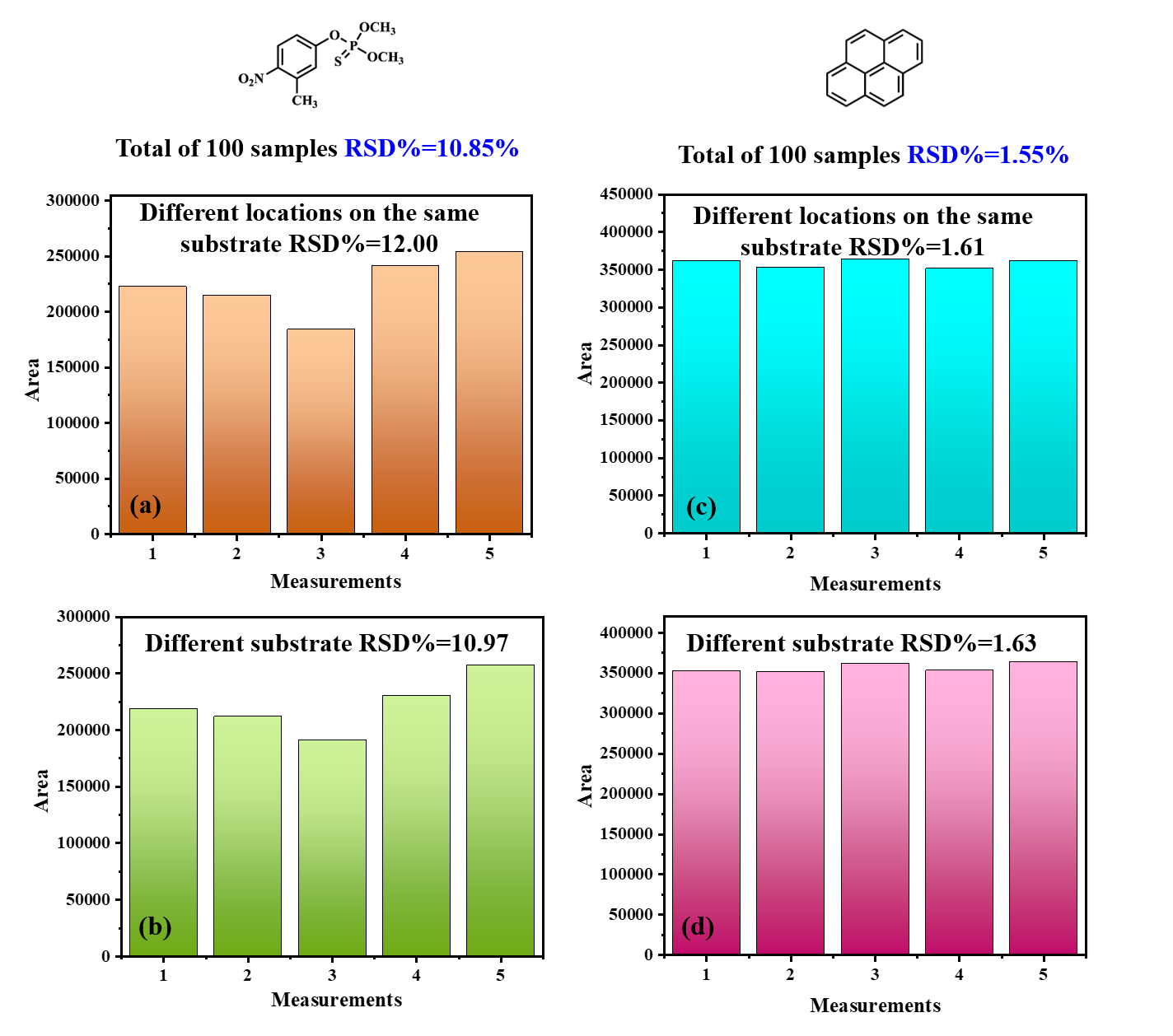


**Figure S16.** The stability result of pyrene and fenitrothion (50 ppm).

**Table S1.** Analytical performance of SERS for PAHs and OPPs.

| Analytes | Linearity equation | Correlation  coefficient | Linear range | LOD | LOQ |
| --- | --- | --- | --- | --- | --- |
| Anthracene | *y* = 8876.7706 *x* + 21531.0960 | 0.9902 | 0.36 ~ 4.00 ^a^ | 0.11 ^a^ | 0.36 ^a^ |
|  | *y* = 48.6051 *x* + 1481.4101 | 0.9946 | 24.52 ~ 500 ^b^ | 7.35 ^b^ | 24.52 ^b^ |
| Pyrene | *y* = 187933.2532 *x* + 13005.7091 | 0.9756 | 0.03 ~ 0.48 ^a^ | 0.01 ^a^ | 0.03 ^a^ |
|  | *y* = 705.9560 *x* + 15396.5243 | 0.9914 | 11.84 ~ 500 ^b^ | 3.55 ^b^ | 11.84 ^b^ |
| Naphthalene | *y* = 242.2134 *x* + 239898.1968 | 0.9593 | 94.72 ~ 980 ^a^ | 28.42 ^a^ | 94.72 ^a^ |
|  | *y* = 320.1404 *x* + 11445.5739 | 0.9937 | 7.8~500 ^b^ | 5.20 ^b^ | 17.32 ^b^ |
| Fenitrothion | *y* = 731.8367 *x* + 198.85.7671 | 0.9817 | 124 ~ 980 ^a^ | 37.20 ^a^ | 124 ^a^ |
|  | *y* = 433.5230 *x* + 10726.3071 | 0.9948 | 7.8 ~ 500 ^b^ | 2.91 ^b^ | 9.71 ^b^ |
| Profenofos | *y =* 35974.0839*x +*19563.1925 | 0.9734 | 0.01 ~ 2.00 ^a^ | 0.01 ^a^ | 0.04 ^a^ |
|  | *y* = 5371.7071*x* + 8375.5313 | 0.9986 | 3.9 ~ 500 ^b^ | 1.21 ^b^ | 4.03 ^b^ |
| Omethoate | *y =* 105.1821 *x +* 25463.2813 | 0.9530 | 50.07 ~ 640 ^a^ | 15.02 ^a^ | 50.07 ^a^ |
|  | *y* = 3733.0802 *x* + 118969.1448 | 0.9909 | 15.29 ~ 500 ^b^ | 4.59 ^b^ | 15.29 ^b^ |
| Isocarbophos | *y =* 69.9264 *x +* 25663.3419 | 0.9769 | 98.27 ~ 980 ^a^ | 29.48 ^a^ | 98.27 ^a^ |
|  | *y =* 961.7860 *x –* 335650.2635 | 0.9761 | 34.77 ~ 500 ^b^ | 10.43 ^b^ | 34.77 ^b^ |

^a^ Represented the concentration unit in ppb.

^b^ Denoted the concentration unit in ppm.
